# A covalent inhibitor targeting the papain-like protease from SARS-CoV-2 inhibits viral replication†

**DOI:** 10.1039/d3ra00426k

**Published:** 2023-04-04

**Authors:** Hesong Han, Albert Vallejo Gracia, Joachim J. Røise, Lydia Boike, Kristoffer Leon, Ursula Schulze-Gahmen, Michael R. Stentzel, Teena Bajaj, Dake Chen, I.-Che Li, Maomao He, Kamyar Behrouzi, Zahra Khodabakhshi, Daniel K. Nomura, Mohammad R. K. Mofrad, G. Renuka Kumar, Melanie Ott, Niren Murthy

**Affiliations:** a Department of Bioengineering, University of California at Berkeley Berkeley CA USA nmurthy@berkeley.edu; b Gladstone Institute of Virology, Gladstone Institutes San Francisco CA USA melanie.ott@gladstone.ucsf.edu; c Department of Medicine, University of California San Francisco CA USA; d Department of Chemistry, University of California Berkeley CA USA; e Innovative Genomics Institute Berkeley CA USA; f Molecular Cell Biomechanics Laboratory, Departments of Bioengineering and Mechanical Engineering, University of California Berkeley CA USA; g Molecular Biophysics and Integrative Bioimaging Division, Lawrence Berkeley National Laboratory Berkeley USA; h Novartis-Berkeley Center for Proteomics and Chemistry Technologies Berkeley CA USA; i Department of Molecular and Cell Biology, University of California Berkeley CA USA; j Department of Nutritional Sciences and Toxicology, University of California Berkeley CA USA; k Chan Zuckerberg Biohub San Francisco CA USA; l Graduate Program of Comparativ Biochemistry, University of California at Berkeley Berkeley CA USA

## Abstract

Covalent inhibitors of the papain-like protease (PLpro) from SARS-CoV-2 have great potential as antivirals, but their non-specific reactivity with thiols has limited their development. In this report, we performed an 8000 molecule electrophile screen against PLpro and identified an α-chloro amide fragment, termed compound 1, which inhibited SARS-CoV-2 replication in cells, and also had low non-specific reactivity with thiols. Compound 1 covalently reacts with the active site cysteine of PLpro, and had an IC50 of 18 μM for PLpro inhibition. Compound 1 also had low non-specific reactivity with thiols and reacted with glutathione 1–2 orders of magnitude slower than other commonly used electrophilic warheads. Finally, compound 1 had low toxicity in cells and mice and has a molecular weight of only 247 daltons and consequently has great potential for further optimization. Collectively, these results demonstrate that compound 1 is a promising lead fragment for future PLpro drug discovery campaigns.

## Abstract 2

Compound 1 is a covalent inhibitor of SARS-CoV-2 PLpro that inhibits viral replication and has low non-specific reactivity with thiols.

## Graphical abstract

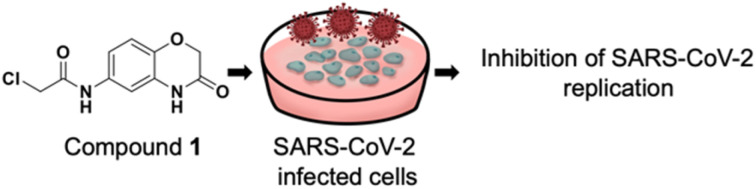

The severe acute respiratory syndrome coronavirus 2 (SARS-CoV-2) has caused catastrophic levels of death and disease and effective treatments for it are urgently needed. Small molecule therapeutics have great potential for combatting SARS-CoV-2 due to their low cost of production, shipping, and storage, and their ability to be self-administered. In addition, small molecules generally work on regions of the virus that are not targeted by vaccines and will most likely be active against vaccine-resistant strains. Despite great effort, remdesivir, molnupiravir and paxlovid, which consists of a co-regiment of nirmatrelvir and ritonavir, are still the only FDA small molecule drugs approved for treating SARS-CoV-2 and have only had a marginal clinical impact.^1,2^ Thus, despite significant efforts, there is still a great need for the development of drugs that can effectively treat SARS-CoV-2.

The papain-like protease (PLpro) from SARS-CoV-2 is an attractive target for developing small molecule drugs. PLpro plays an essential role in viral replication and its inhibition prevents viral replication in cells.^3–7^ In addition, PLpro suppresses the production of interferons, which are essential for mounting an immune response against SARS-CoV-2. PLpro cleaves the peptide sequence LxGG, which is present in 3 sites in the immature SARS-CoV-2 viral poly-protein. PLpro catalyzes the release of three non-structural proteins, termed nsp1, nsp2, and nsp3 from the immature viral poly-protein. Nsp1, nsp2, and nsp3 play critical roles in viral replication, and inhibition of PLpro blocks SARS-CoV-2 replication in cells.^3,5,8^ PLpro also cleaves host proteins that contain the sequence RLRGG, which is present in several ubiquitin (Ub) and ubiquitin-like proteins (UbL), such as interferon-induced gene 15 (ISG15) proteins. PLpro has significant deSIGylating and deubiquitinating activities and inhibition of PLpro induces the production of interferons by virally infected cells, which should lead to an enhanced immune response against the virus.^9^ Consequently, there is great interest in developing inhibitors against PLpro from SARS-CoV-2.^7,8^

PLpro is a cysteine protease with a catalytic triad composed of histidine, cysteine, and glutamic acid, and has 83% sequence homology to PLpro from SARS and also has structural similarities to the de-ubiqinating enzyme UBL22.^3,5,8,10^ Several crystal structures of PLpro have been solved, and these studies have revealed that it has an *N*-terminal ubiquitin-like (Ubl) domain and a C-terminal catalytic domain, which has a ubiquitin-specific protease (USP) domain. The catalytic domain has an open-right-hand fold, with thumb, palm, and fingers subdomains, and a structural zinc ion is located at the tip of the fingers domain. PLpro binds gly–gly in the first two positions of its peptide binding site and does not have a well-defined binding pocket near its active site, in contrast to other proteases that need to accommodate peptides with larger side chains. PLpro is considered to be a challenging protein to drug due to its ill-defined binding pocket. Progress towards developing PLpro inhibitors has been much slower than for Mpro inhibitors, despite the fact they are both cysteine proteases.^4–6,8,11–14^

Several HTS screens have been performed against PLpro from SARS-CoV-2 and SARS, and these studies have generated only a few classes of pharmacophores that can inhibit PLpro and viral replication in cells.^10,15,16^ The compound GRL-0617 and its derivatives are the best characterized class of PLpro inhibitors. GR-0617 was originally identified in 2008 by the Mesecar laboratory, who identified it by performing a 50 000-molecule screen on PLpro from SARS-CoV. GRL-0617 inhibited SARS-CoV PLpro with an IC_50_ of 0.6 μM and inhibited viral replication in cells with an EC_50_ of 14.5 μM.^10^ This compound was further optimized and its variants had IC_50_ values as low as 150 nM and were able to inhibit SARS-CoV replication in cells with an EC_50_ of 5 μM.^17^ GRL-0617 also inhibits PLpro from SARS-CoV-2 and viral replication in cells, with IC_50_ in the micromolar range, and shows moderate antiviral activity against SARS-CoV2 in mice after oral delivery.^23^ GRL-0617 has been further optimized against PLpro from SARS- CoV-2 *via* structure based drug design strategies, and its derivatives inhibit PLpro with nanomolar efficacy *in vitro* and also inhibit viral replication in cells efficiently.^15^ Innovative methods for screening PLpro inhibitors in cells have also been developed, based on a FlipGFP reporter assay, which have generated additional promising GRL-0617 based inhibitors that were able to inhibit viral replication in cells.^24^

HTS screens on PLpro from SARS-CoV-2 have also generated promising lead pharmacophores that are not based upon GLR-0617. For example, Yuan *et al.* screened a 50 000 large compound library and identified a new class of PLpro inhibitors, based upon the fragment 5-oxo-1-thioxo-4,5-dihydro[1,3] thiazolo[3,4-*a*]quinazoline-3-carboxamide, which inhibited PLpro from multiple coronaviruses and also SARS CoV-2 viral replication in hamsters and MERS in mice.^23^ These pioneering studies demonstrate the great potential of PLpro inhibitors based on new chemical scaffolds.^11,15,16,18^

PLpro is a cysteine protease with a nucleophilic cysteine in its active site, which can also be targeted with covalent inhibitors.^11,19^ Several electrophiles that can inhibit PLpro *in vitro* have been identified. For example, α-chloro ketone based inhibitors have been identified that can inhibit PLpro with IC_50_s in the micromolar range.^19^ In addition, peptide inhibitors with acrylate warheads, termed VIR250 and VIR251, were also able to inhibit PLpro, *via* alkylation of the active site cysteine, with IC_50_s in the 20–30 μM range.^7^ In addition, VIR250 and VIR251 had high specificity for PLpro and inhibited it without interfering with the deubiquinating activity of UCH-L3, an enzyme with close structural homology to PLpro. Crystal structures of VIR250 and VIR251 with PLpro have been solved and this structural data should enable their further optimization. In addition to these promising lead fragments, a covalent inhibitor of PLpro has also been disclosed based upon the GRL-0617 compound that contained a fumarate ester warhead. These GRL-0617 based electrophiles inhibited PLpro with nanomolar IC50s and were also able to protect Vero-6 cells from the cytopathic effects of SARS CoV-2 at micromolar concentrations.^25^ These studies suggest that covalent inhibitors may be able to overcome the druggability problems associated with PLpro. However, there are still only a few examples of covalent PLpro inhibitors and there is a great need for the development of new covalent PLpro inhibitors.

Fragment-based electrophile screening is an attractive method for developing covalent inhibitors and has been used to develop inhibitors against a wide range of cysteine-containing enzymes.^20,21^ Fragment-based electrophile screens can identify classes of electrophiles that react with an enzyme's active site and can also identify chemical fragments that have affinity for the active site of a protein.^5^ A potential limitation of fragment-based electrophile screening is the large variability in the non-specific reactivity of the electrophiles in an electrophile library. Non-specific reactivity can be a major limitation for electrophile based inhibitors. Electrophiles with high non-specific reactivity will also have low IC_50_s and will emerge as promising hits, even though they have little specificity for their target enzyme. However, the confounding effects of electrophile non-specific reactivity can potentially be accounted for by measuring their reactivity with glutathione.

In this report, we screened an 8000-compound electrophile fragment library against PLpro from SARS-CoV-2 and also determined the non-specific reactivity of our top hits against glutathione. From this screen, we identified a covalent inhibitor based upon an α-chloro amide fragment, termed compound 1, which could inhibit SARS-CoV-2 replication in cells and had low non-specific reactivity with thiols (see Fig. 1). Compound 1 had a *t*_1/2_ of 1456 minutes in the presence of 5 mM glutathione, and was 1–2 orders of magnitude lower in reactivity than the other α-chloro ketones identified from our screen. Finally, compound 1 had low toxicity in cells and mice and has a molecular weight of only 247 daltons and consequently has great potential for further optimization. Collectively, these results demonstrate that compound 1 is a promising lead fragment for future PLpro drug discovery campaigns.

**Fig. 1:**
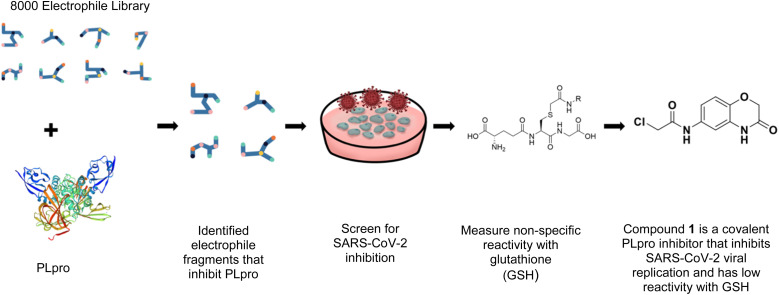
Compound 1 is a covalent inhibitor of PLpro that inhibits SARS-CoV2 viral replication and has low non-specific reactivity with thiols. In this report, we screened an 8000-compound electrophile library for PLpro inhibitors. Promising hits were screened for their ability to inhibit SARS-CoV-2 replication in cells and for their non-specific reactivity with thiols. The α-chloro amide fragment compound 1 inhibited SARS-CoV-2 viral replication in cells, had low non-specific reactivity with thiols, had low toxicity in cells and mice, and has a molecular weight of only 247 daltons. Collectively, these attributes make compound 1 a promising lead fragment for future drug development.

We screened PLpro against an 8000 molecule electrophile library using a fluorescent assay based upon the peptide substrate R-L-R-G-G-AMC (see ESI† for details). The electrophile library contained a variety of weak electrophiles, composed of ureas, acrylamides, α-chloro-amides, epoxides, alkyl halides, boronates, and sulfonyl fluorides. The fragments in this library were 200–400 Da in molecular weight. We initially screened this library at 200 μM, after a 30 minutes pre-incubation with PLpro, and compounds that caused >90% inhibition were selected for further analysis. This initial screen gave approximately 30 hits, consisting of ureas, α-chloro-amides, sulfonyl fluorides, and vinyl sulfones. We repurchased these hits, further evaluated their PLpro inhibitory activity, and identified several fragments that reproducibly inhibited PLpro activity. The structures of 6 representative hits and their IC_50_'s are shown in Table 1; their IC_50_'s ranged from 300 nM to 20 μM. These 6 hits were chosen for further exploration because multiple similar hits from each fragment class were identified. The cell culture toxicity of these hits were also determined and 5/6 of them had CC_50_s > 500 μM.

**Table 1:** The IC_50_s and CC_50_s of the top 6 hits from an electrophile screen performed to identify PLpro inhibitors^a^

Entry	Structure	IC_50_/μM	MTT CC_50_/μM
1	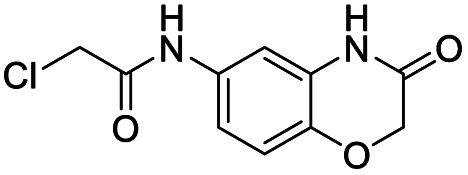	18	>500
2	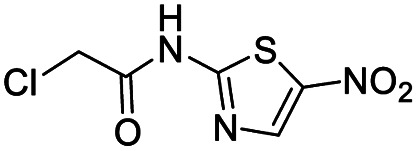	13	>500
3	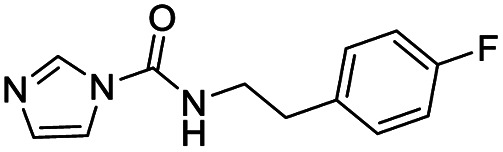	0.3	62.5
4	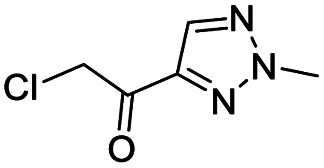	2.5	>500
5	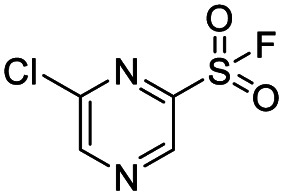	1.7	>500
6	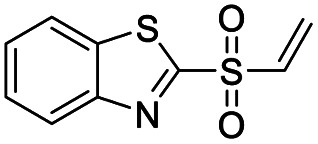	1.0	>500

a Listed in Table 1 are the top 6 hits from the PLpro electrophile screen. 4 different electrophile classes were able to inhibit PLpro activity, α-chloro amides, ureas, sulfonyl fluorides and vinyl sulfones. The IC_50_ values of the electrophile hits varied from 18 μM to 450 nM, and 5/6 of them had CC_50_'s > 500 μM against Calu-3 cells.

The six compounds that were found to inhibit PLpro were tested for their ability to inhibit SARS-CoV-2 infection in cell lines, specifically the human lung epithelial Calu-3 cell line and monkey kidney Vero E6 cells. Cells were incubated with PLpro inhibitors at various concentrations and infected with SARS-CoV-2 (US/WA1/2020 isolate) at a multiplicity of infection (MOI) of 0.1 (Calu-3) or 0.05 (Vero E6) for 72 hours and analysed for SARS-CoV-2 replication *via* staining of intracellular double stranded RNA (dsRNA) and RT-qPCR analysis of viral RNA in the supernatant. Cells infected with SARS-CoV-2 and treated with either DMSO or remdesivir served as negative and positive controls, respectively. Compound 1 was the only PLpro inhibitor identified that blocked SARS-CoV-2 replication in both cell lines and had an IC_50_ < 20 μM for inhibiting viral replication (Fig. 2A–D).

**Fig. 2:**
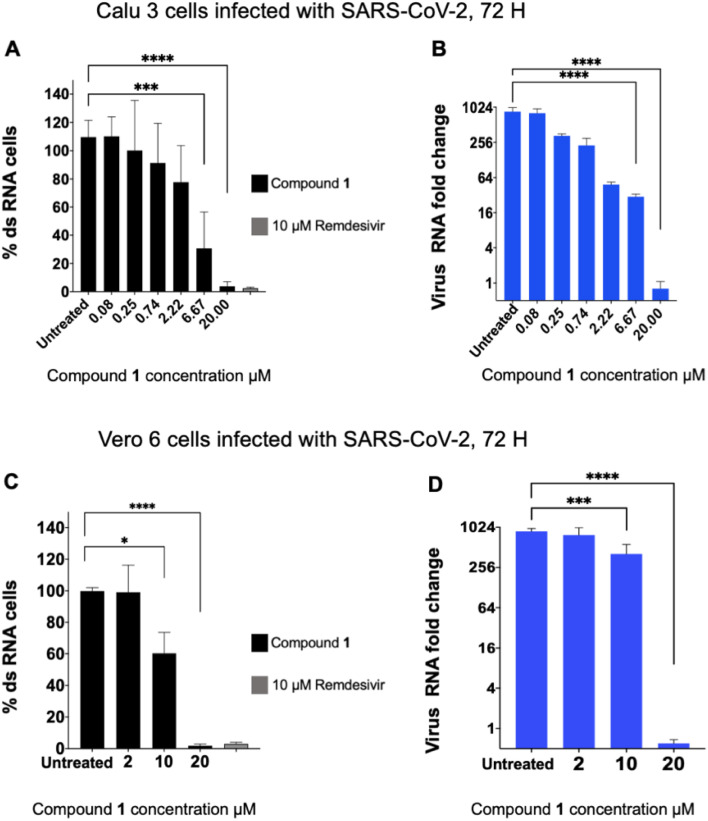
Compound 1 inhibits SARS-CoV-2 viral replication. Compound 1 was incubated with cells and then infected with SARS-CoV-2, and the levels of intracellular double stranded RNA (dsRNA) or viral RNA in the supernatant, in Calu-3 (A and B) or Vero E6 (C and D) cells, were measured. Percent dsRNA was determined by normalizing against DMSO-treated infected cells. Levels of viral RNA in the supernatant were determined by RT-qPCR (*n* = 8 for Calu-3 cells, *n* = 5 for Vero6 cells). Compound 1 inhibited viral replication in both cell lines.

We investigated the cytotoxicity of compound 1 in Calu-3 cells and determined its maximal tolerated dose (MTD) in mice. Compound 1 was incubated with Calu-3 cells at various concentrations from 10 μM to 500 μM for 48 hours and the cell viability was determined with the MTT assay and compared against DMSO treated controls. In addition, compound 1 was given to mice *via* intraperitoneal injection, and weight loss was monitored. Compound 1 had very little toxicity in cells and mice, with a CC_50_ > 500 μM in cells and an MTD of >1 gram per kg (see ESI Fig. S2†).

Compound 1 contains an α-chloro amide fragment which can potentially react with the active site cysteine (Cys111) of PLpro.^5^ We performed experiments with compound 1 to investigate if it irreversibly alkylated Cys111 of PLpro. PLpro was mixed with 50 μM of compound 1 for 1 hour, which is approximately three times its IC_50_, after which it was diluted to 500 nM and assayed for PLpro inhibition. Fig. 3A demonstrates that pre-incubation of compound 1 with PLpro caused complete inhibition of PLpro, even after dilution to 500 nM, which is 36-fold lower than the IC_50_ of compound 1. This supports the assumption that compound 1 is an irreversible inhibitor of PLpro. In addition, we performed mass spectrometry analysis of PLpro after incubation with compound 1, and found that compound 1 reacts with Cys111 in the active site of PLpro (see ESI Fig. S5†). Molecular dynamics simulation of compound 1 with PLpro suggested that it fits into the active site of PLpro and its α-chloro-amide appears to form a hydrogen bond with the amide NH of tyrosine 112.

**Fig. 3:**
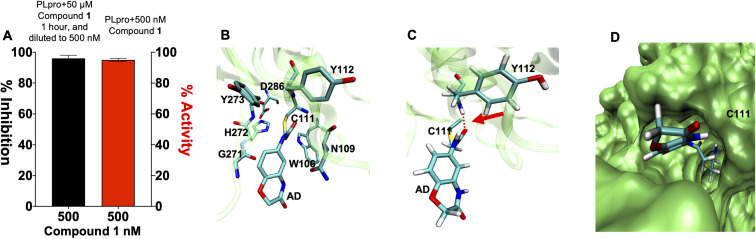
Compound 1 is an irreversible inhibitor of PLpro and alkylates the active site cysteine. (A) Compound 1 was pre-incubated with PLpro at a 50 μM concentration for 1 hour and then diluted down to 500 nM. PLpro activity was measured and compared against PLpro incubated with 500 nM of compound 1. Compound 1 causes complete inhibition of PLpro after pre-incubation at 50 μM (black line) and dilution to 500 nM, in contrast, compound 1 causes no inhibition at 500 nM (red line), without pre-incubation at 50 μM. All error bars represent the standard error of the mean for *n* = 3 replicates. (B–D) Molecular dynamics simulation of compound 1 binding PLpro (B). Compound 1 makes a covalent bond with CYS111 and interacts with residues in the active site. The interaction of compound 1 with TYR112 is shown. (C) Compound 1 is close to TYR112 and makes a hydrogen bond; see arrow. (D) Surface rendering of the active site of PLpro with compound 1. Compound 1 goes inside the active pocket and interacts with residues in the active site.

We synthesized a library of 15 derivatives of compound 1, following the synthetic scheme shown in the ESI (Fig. S4†). The ability of the compound 1 derivatives to inhibit PLpro was investigated. 2 hits were identified from this small screen, termed compounds 19 and 20, which had a lower PLpro inhibition IC_50_ than compound 1 (see Fig. 4). Compounds 19 and 20 were approximately 3-4-fold more active than compound 1. All of the other compounds synthesized were either inactive or had much lower activities than compound 1. Compound 1 thus tolerates modifications at the 7′ position, and electron withdrawing groups appear to increase its efficacy. Compounds 19 and 20 were also investigated for their abilities to inhibit viral growth in cells, and they had no inhibitory activity against SARS-CoV-2 (see ESI Fig. S1†). Nonetheless, this study shows that compound 1 can be structurally modified at its 7′ position and opens up a viable path for future optimization studies.

**Fig. 4:**
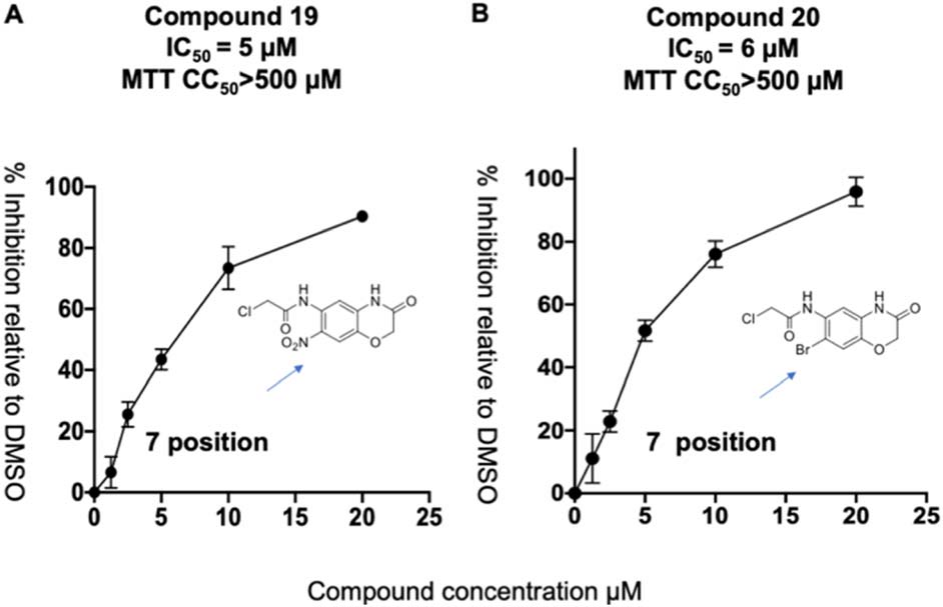
Compound 1 tolerates modification at its 7′ position. (A and B) A preliminary SAR emerged from the compound 1 derivatives, which demonstrates that substituents at the 7′ position are well tolerated and that electron withdrawing groups at this position can increase the activity of this class of PLpro inhibitors. Compounds 19 and 20 had IC_50_'s of 5 and 6 μM and have IC_50s_ that are approximately 3 times lower than compound 1's.

Compound 1 has a relatively high PLpro inhibition IC_50_ (18 μM), yet it performed better than numerous other fragments identified from our screen, which had IC_50_'s as low as 300 nM. For weak electrophiles, non-specific reactivity with thiols can be a major limitation. Several of the hits identified from our screen can be categorized as promiscuous frequent hitters, and their lack of activity in cells is presumably due to their non-specific reactivity with glutathione.^21,22^ The thiol reactivity of electrophiles can vary by orders of magnitude and we therefore determined the reaction half-lives of compounds 1–4, 6, 19 and 20, with GSH to determine if non-specific reactivity with thiols could explain the high cellular efficacy of compound 1 (see ESI Fig. S3†). The *t*_1/2_ of compounds 1–4 and 6, 19 and 20, in the presence of 5 mM GSH is shown in Table 2. The glutathione reactivity of the 7 inhibitors varied by orders of magnitude, and they had a *t*_1/2_ ranging from 2 minutes to over 1400 minutes.

**Table 2:** The glutathione reactivity of electrophile based PLpro inhibitors varies by three orders of magnitude

Entry	Structure	*t*_1/2_ per min
		5 mM GSH
1	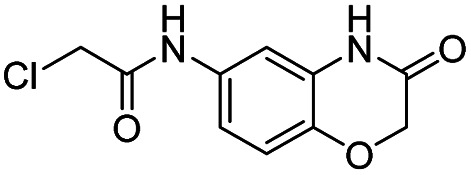	1456
2	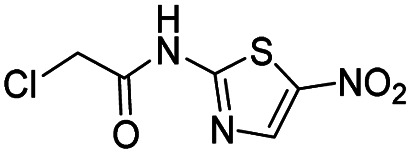	76
3	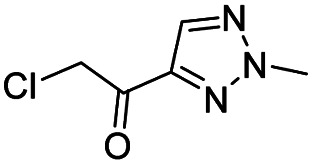	2.8
4	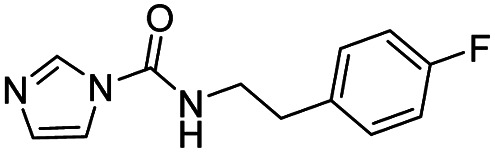	13.4
6	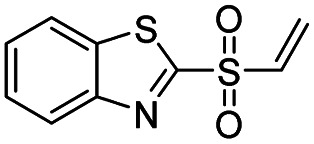	<1
19	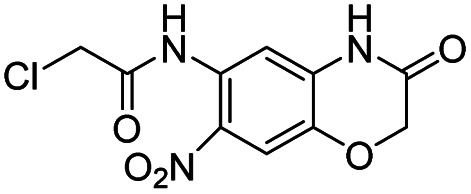	144
20	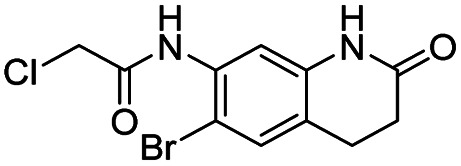	330

The GSH reactivity explains why the most effective compounds, based upon IC_50_, were not active in cells. For example, the most effective PLpro inhibitor, compound 3, had an IC_50_ of 300 nM, and was not effective in cells, however it had a *t*_1/2_ of 2.8 minutes in the presence of 5 mM GSH, and this presumably led to its lack of efficacy in cells. In contrast, compound 1 reacted relatively slowly with GSH, and had a *t*_1/2_ of 1456 minutes. In addition, compound 1 was able to inhibit PLpro in the presence of 5 mM glutathione or 5 mM DTT (see ESI Fig. S6†), and can inhibit PLpro under physiologic thiol concentrations. Compound 1 also did not inhibit the Mpro protease from SARS CoV-2 (see ESI Fig. S5†). Compound 1 had a stability that was comparable to phenyl acrylamide-based electrophiles, which are present in clinically approved drugs, such as Afatinib, Sotorasib and Osimertinib. For example, phenyl acrylamide has a reaction half life of 179 minutes with glutathione at 5 mM GSH.^22^ We also measured the *t*_1/2_ of compounds 19 and 20, which were structural analogues of compound 1 and had lower IC_50_ values than compound 1, but did not inhibit viral replication (see ESI Fig. S1†). Compounds 19 and 20 both reacted with GSH faster than compound 1, suggesting that the GSH *t*_1/2_ may play a very important role in determining the efficacy of covalent inhibitors.

In summary, in this report we present a covalent PLpro inhibitor that can inhibit SARS-COV-2 replication in cells.^19^ Compound 1 was not the most effective inhibitor identified from our screen, however it was the only compound that inhibited viral replication in cells. Compound 1's effectiveness in cells appears to be due to its low non-specific reactivity with thiols. Finally, compound 1 had low toxicity in cells and mice and has a molecular weight of only 247 daltons and consequently has great potential for further optimization. Collectively, these results demonstrate that compound 1 is a promising lead fragment for future PLpro drug discovery campaigns.

## Ethical statement

The animal study was reviewed and approved by the University of California, Berkeley Animal Care and Use Committee (ACUC) and Laboratory Animal Care(OLAC) AUP-2019-04-12046. All the mice were purchased from Jackson laboratory (Maine, USA).

## Conflicts of interest

The authors have no conflicts to declare.

## Supplementary Material

RA-013-D3RA00426K-s001
