# Supplementary material for: A covalent inhibitor targeting the papain-like protease from SARS-CoV-2 inhibits viral replication
Source: RSC Adv. 2023 Apr 4;13(16):10636–41. doi: 10.1039/d3ra00426k (PMC10072198; doi:10.1039/d3ra00426k)
Supplement: RA-013-D3RA00426K-s001 [file RA-013-D3RA00426K-s001.pdf]

Electronic Supplementary Material (ESI) for RSC Advances.
This journal is © The Royal Society of Chemistry 2023

## Materials and Methods

All compounds were purchased from Sigma Aldrich (St. Louis, MO, USA), Fisher Scientific (Hampton, NH, USA), or VWR (Radnor, PA, USA). The 8000-compound covalent fragment library was purchased from Enamine (Kyiv, Ukraine). The reported 1H NMR and 13C NMR were recorded on a Bruker Avance 400 console with an Oxford Instruments 9.4 T magnet at 400 and 101 MHz respectively at the College of Chemistry NMR Facility at UC Berkeley. Compound purity was assessed by $^{1}$H-NMR spectroscopy. Mass spectra were collected at the QB3/Chemistry Mass Spectrometry Facility at UC Berkeley. Absorbance was measured on a Tecan infinite M200 plate reader (Männedorf, Switzerland). High performance liquid chromatography data was collected on a modular HPLC instrument from Shimadzu (Kyoto, Japan) assembled from a CBM-40 System Controller, two LC-20AR pumps, an ASI 500uL mixer, a DGU-405 degasser, and a CTO-40C oven with an XBridge C18 5µm column (4.6x150mm) and an SPD-M20A UV/Vis detector.

## Protein expression and purification

A PLpro expression plasmid in a 2TB vector was transformed into the *E.coli* BL21 (DE3) strain. The transformed cells were cultured in LB medium at 37 °C with 100 µg/ml ampicillin till it reached an optical density of 1.0 and 100 mM of Isopropyl-β-D-thiogalactopyranoside (IPTG) was added. The bacterial cells were harvested after 16°C overnight and the cell pellet was resuspended in lysis buffer [200 mM NaCl, 5%(v/v) glycerol, 20 mM Tris, 20 mM imidazole, 0.1% (v/v) Triton-X 100, pH 7.5]. The resuspended solution was sonicated for 5 minutes (10 second on, 30 second off) and centrifuged at 35,000 g for 1 hour at 4 °C. The clarified supernatant was loaded to a GE HisTrap FF column (GE Healthcare Life Sciences) and washed with binding buffer [500 mM NaCl,20 mM imidazole, 20 mM Tris,pH7.5] to eliminate unspecific protein. Bound protein was eluted with elution buffer [500 mM NaCl,500 mM imidazole, 20 mM Tris, 5%(v/v) glycerol, pH7.5]. The His6-tag was cleaved by TEV protease treatment at 1:40 protease: protein ratio in dialysis buffer [150 mM NaCl, 10%(v/v) glycerol, 20 mM Tris,10 mM TCEP, pH7.5] at 4 °C for overnight. The overnight solution was loaded to GE HisTrap FF column again to remove cleaved His-tag and TEV protease. The protein sample was further purified using GE Superdex-200-og SEC column equilibrated with 150 mM NaCl, 20 mM Tris,10 mM DTT.

## Fluorescence based PLpro activity assays

A PLpro activity assay was conducted with the peptide substrate R-L-R-G-G-AMC (Bachem). PLpro was added into black, flat-bottom 96-well plates at a final concentration of 50 nM with reaction buffer [150 mM NaCl, 20 mM HEPES, 0.1mg/mL BSA, pH 7.5], and mixed with varying concentration of inhibitors (0-200 µM) for 20 min prior to substrate addition at room temperature. The reaction was initiated by mixing the enzyme solution with R-L-R-G-G-AMC, to generate a final concentration of 50 µM, and incubated for 20 min at room temperature. The reaction was ended by the addition of 1µL of aqueous citric acid, to generate a final concentration of 10mM. The PLpro activity was detected by measuring the fluorescence emission intensity $\lambda_{excitation}$=360 nm; $\lambda_{emission}$=460nm.

## Cell MTT assay

A cell viability assay was performed to assess the cytotoxicity of compounds against Calu 3 cells. Calu 3 cells (10,000 cells/well) were seeded in 96-well plates and allowed to grow in DMEM supplemented with 10% (v/v) FBS, 0.05 mg/mL penicillin G and 80 µg/mL streptomycin and incubated at 37°C with 5% $CO_2$ for 24 h. Then, cells were treated with various concentrations of compounds. Control cells were added with equivalent volume of fresh media. After incubation for 24 h, a final concentration of 1 µg/ml Resazurin was added to each well and incubated for 4 h under cell culture conditions. The fluorescence of the plate was measured with a plate reader with Ex=540 nm, Em=590 nm. The cell growth inhibition was calculated.

## SARS CoV-2 infection of cells

Vero 6 cells or Calu 3 cells were seeded in 96-well plates (40,000 cells/well) and allowed to grow in DMEM supplemented with 10% (v/v) FBS, 0.05 mg/mL penicillin G and 80 µg/mL streptomycin and incubated at 37°C with 5% $CO_2$ for 24 h. Cells were treated with compounds for 1 hour, infected with SARS-CoV-2 at a MOI of 0.05 for Vero 6 and 0.1 for Calu 3 for 72 hours. Remdesivir was included as a positive control. A 150 µL of supernatant was collected, fixed with 500 µL Trizol (in deep 96-well plates), and stored at -80°C for RNA extraction to determine viral RNA levels by qRT-PCR. Cells were then washed with PBS and fixed with 4% PFA, fixed cells were permeabilized and stained with a dsRNA antibody to detect viral replication.

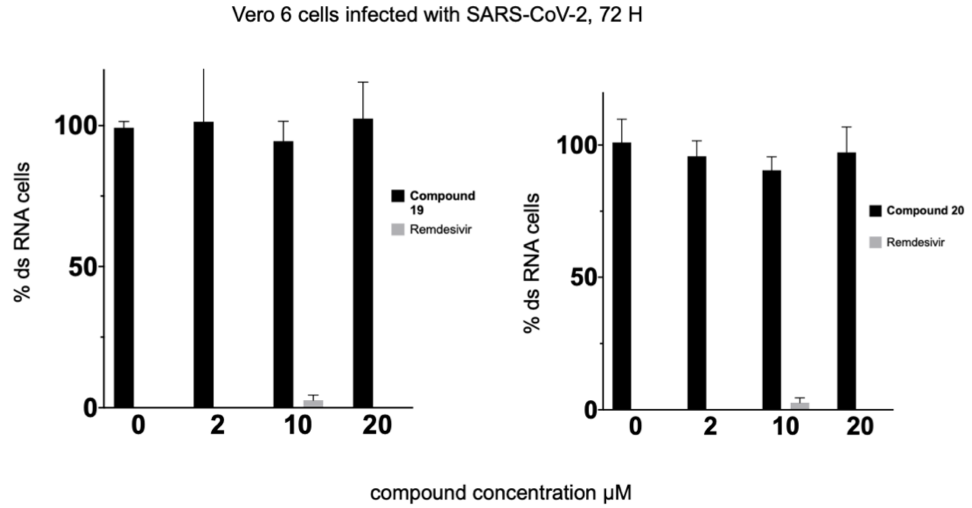


**Figure S1.** Levels of intracellular double stranded RNA in Vero E6 cells infected with SARS-CoV-2 in the presence of various concentrations of compound **19** and compound **20**. Percent dsRNA was determined by normalizing against DMSO-treated infected cells.

## qRT-PCR

100 µL of cell culture fluids were collected and mixed with 300 µL Trizol or RLT buffer. RNA was extracted and purified using the PureLink RNA mini kit (Invitrogen) according to the manufacturer's instructions. RNA was analyzed with a SuperScript One-step qRT-PCR kit to quantify viral RNA copy numbers. The forward primer (*ACAGGTACGTTAATAGTTAATAGCGT*) and reverse primer (*ATATTGCAGCAGTACGCACACA*) were used to amplify the SARS-CoV-2 E RNA.

**Mouse MTD assay**

Ethical statement

The animal study was reviewed and approved by the University of California, Berkeley Animal Care and Use Committee(ACUC) and Laboratory Animal Care(OLAC) AUP-2019-04-12046. All the mice were purchased from Jackson laboratory (Maine, USA)

20 mg/kg of compound **1** was administered to 3 female Balb/c mice (6-8 weeks old) via intraperitoneal injection. The mouse body weight was monitored for 7 days. The results are shown in Figure S2.

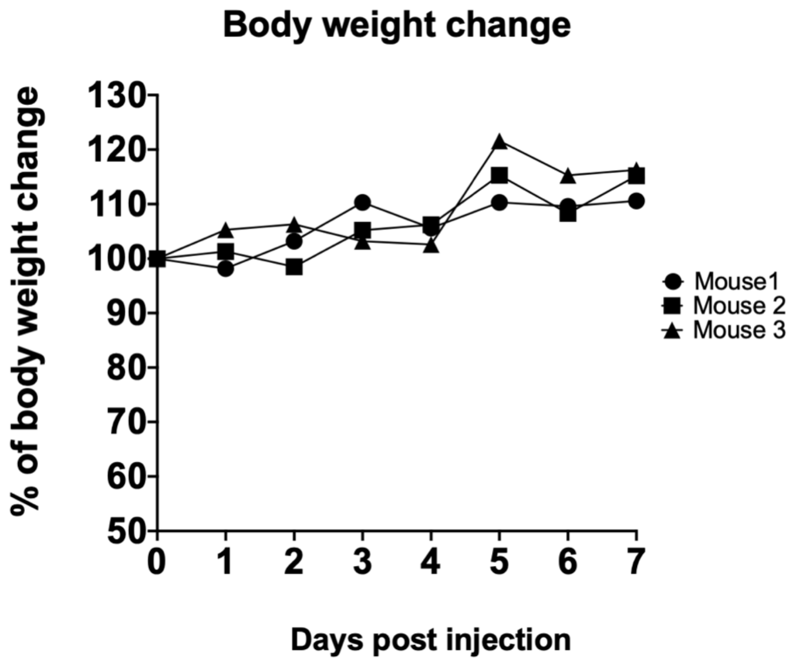


**Figure S2:** Compound **1** has minimal toxicity to mice at a dose of 20 mg/kg.

**Molecular Dynamics Simulation of PLpro with compound 1**

To model the dynamics of compound **1** inside the PLpro active site, we drew the chemical structure of compound **1** in MarvinSketch. OpanBable was used to convert 2D chemical structures

into 3D. The protein structure of SARS-CoV-2 PLpro (PDB: 6W9C) was obtained from RCSB server. The CHARMM36 forcefield was used to simulate protein ligand interactions, the CGENFF server was used to optimize forcefield parameters for compound **1**. Molecular Dynamics (MD) simulation of PLpro and compound **1** interactions were performed with GROMACS. In all cases, we put the protein-ligand complex inside the water box with 1 nm spacing from each side, then we neutralized the system with $Na^+$ and $Cl^-$. In order to model water molecules dynamics, we used the TIP3P forcefield which considers three sites for each molecule. To insert compound **1** with energy minimized structures inside the active site of PLpro, we used 1-ClickDocking online server, which provides final structures of drugs positioned outside of the active site. After getting non-covalent docking results from 1-ClickDocking server, we used VMD to manually put drugs inside the active side near CYS111. Now we can use these new complex structures to model covalent inhibition of PLpro using MD simulation. We used particle mesh Ewald (PME) method to capture long range electrostatic interactions. To equilibrate the system, we did NVT and NPT simulations for 100 ps with V-rescale thermostat and Berendsen barostat. This general MD setup is similar to prior studies that our group has done. The MD production simulations were set to model the dynamics of the system in 30 ns using V-rescale thermostat and Parrinello-Rahman barostat. For the covalent interaction between PLpro CYS111 and ligands, we manually added a thioether between the sulfur atom of CYS111 and compound **1**. Finally, the structures of PLpro combined with compound **1** were visualized with VMD.

**LC-MS/MS analysis of PLpro incubated with compound 1**

Purified PLpro (20 µg) in 100 µL buffer of 150 mM NaCl, 20 mM HEPES, 5 mM TCEP was incubated for 30 min at room temperature with 10 µM Compound **1**. The sample was precipitated by addition of 25 µL of 100% (w/v) trichloroacetic acid before cooling to −80 °C for 1 h. Following incubation, the sample was centrifuged at max speed for 10 min at 4 °C. The supernatant was carefully removed and the sample washed 3X with ice-cold 0.01 M HCl/90% acetone solution. The pellet was then resuspended in 4 M urea containing 0.1% Protease Max (Promega, V2071) and diluted in 40 mM ammonium bicarbonate buffer. 10 mM TCEP was then added and the samples were incubated at 60 °C for 30 min. Next, 12.5mM iodoacetamide was added and the sample incubated for another 30 minutes at 37 °C. The sample was then diluted 50% with PBS

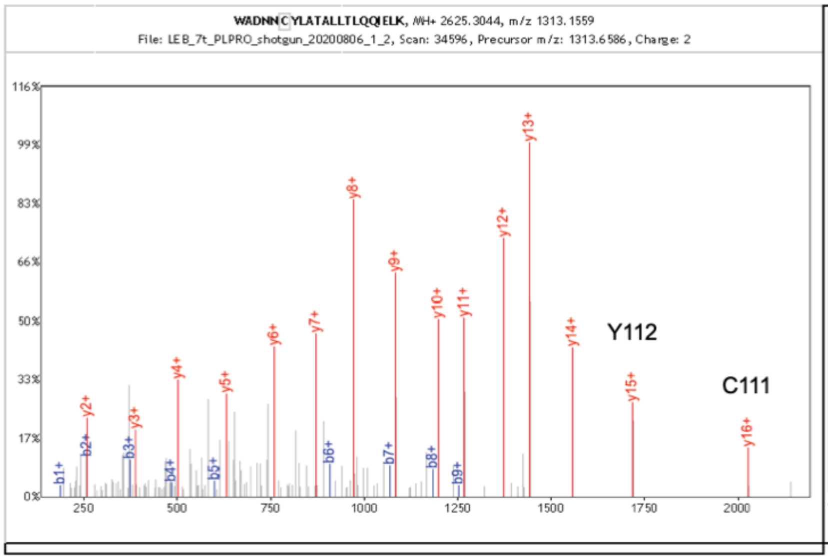


| b+ | # | Seq | # | y+ |
|---|---|---|---|---|
| 187.0866 | 1 | W | 21 | |
| 258.1237 | 2 | A | 20 | 2439.2251 |
| 373.1506 | 3 | D | 19 | 2368.1880 |
| 487.1936 | 4 | N | 18 | 2253.1611 |
| 601.2365 | 5 | N | 17 | 2139.1182 |
| 908.3019 | 6 | C | 16 | 2025.0752 |
| 1071.3652 | 7 | Y | 15 | 1718.0098 |
| 1184.4493 | 8 | L | 14 | 1554.9465 |
| 1255.4864 | 9 | A | 13 | 1441.8625 |
| 1356.5341 | 10 | T | 12 | 1370.8253 |
| 1427.5712 | 11 | A | 11 | 1269.7777 |
| 1540.6552 | 12 | L | 10 | 1198.7406 |
| 1653.7393 | 13 | L | 9 | 1085.6565 |
| 1754.7870 | 14 | T | 8 | 972.5724 |
| 1867.8710 | 15 | L | 7 | 871.5247 |
| 1995.9296 | 16 | Q | 6 | 758.4407 |
| 2123.9882 | 17 | Q | 5 | 630.3821 |
| 2237.0723 | 18 | I | 4 | 502.3235 |
| 2366.1149 | 19 | E | 3 | 389.2395 |
| 2479.1989 | 20 | L | 2 | 260.1969 |
| | 21 | K | 1 | 147.1128 |

[Click] to move table

Variable Modifications:
C: 204.05619

before sequencing grade trypsin (1 µg per sample, Promega, V5111) was added for an overnight incubation at 37 °C. The following day, the sample was centrifuged at 13,200 rpm for 30 min, and the supernatant transferred to a new tube and acidified to a final concentration of 5% formic acid and stored at −80 °C until mass spectrometry analysis.

**Determination of GSH $t_{1/2}$ values**

To a solution of glutathione (190uL, 5.26mM in PBS pH7.4) was added electrophile (10uL, 1mM in DMSO) to give a solution with final concentrations of 5mM GSH and 0.05mM electrophile, and 5% DMSO. The resulting mixture was allowed to incubate at 37°C until quenched at varied timepoints with 10% $H_3PO_4$ (20uL) and stored at -80°C until HPLC injection. GSH $t_{1/2}$ values were then measured as the time taken for the starting electrophile peak to halve in area (@254nm), by finding the $k_{1st}$(obs) value and calculating the $t_{1/2}$ using the equation $t_{1/2}=\ln(2)/k_{1st}(obs)$.

*HPLC method*

Program: MeCN/$H_2O$ + 0.1% TFA, 0-2min: 5% MeCN, 2-15min: gradient to 100% MeCN, 15-17min: 100% MeCN, 17-19min; gradient to 5% MeCN. Flow rate 1.0mL/min.

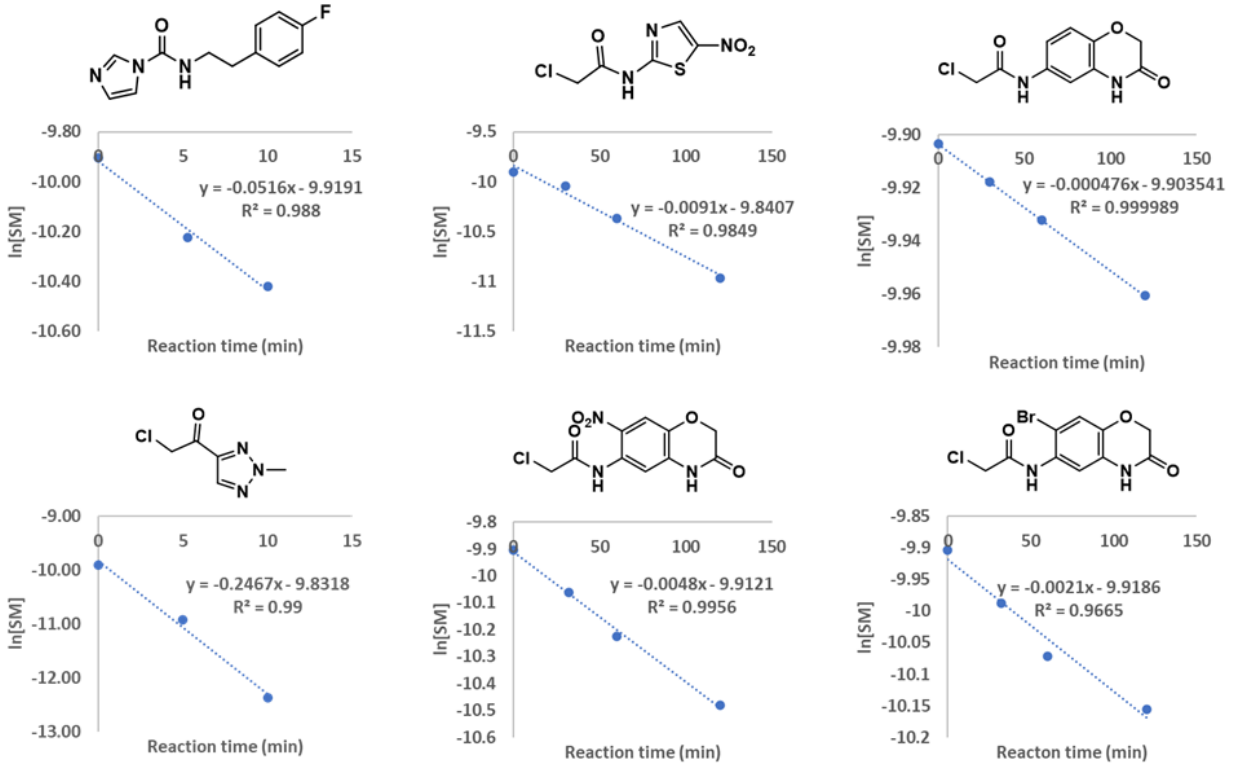


**Figure S3:** Pseudo first-order rate constants for the reaction of electrophile hits (0.05mM) with glutathione (5mM). The plots are given as ln[electrophile] over time (min), to give a linear curve with the $k_{1(obs)}$ as the slope.

## Synthesis Protocols

Compound **1** derivatives and $IC_{50}$ values

| | Structure | $IC_{50}$ (μM) |
|---|---|---|
| 1 | 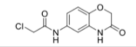 | 18 |
| 7 | 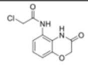 | 100 |
| 8 | 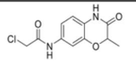 | >100 |
| 9 | 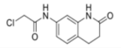 | >100 |
| 10 | 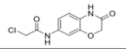 | >100 |
| 11 | 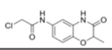 | >100 |
| 12 | 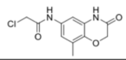 | 12 |
| 13 | 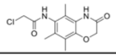 | >100 |
| 14 | 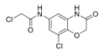 | 9 |
| 15 | 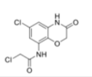 | >100 |
| 16 | 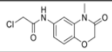 | >100 |
| 17 | 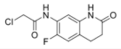 | >100 |
| 18 | 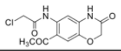 | >100 |
| 19 | 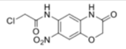 | 5 |
| 20 | 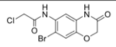 | 6 |

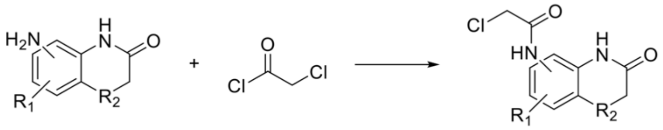

**Figure S4:** Synthesis of *alpha*-chloro amides. Reagents and conditions: 0.63 mmol amine and 0.69 mmol chloroacetyl chloride in DCM for 4 hours.

**Preparation of *alpha*-Chloro Amides: General Procedure A:** A 5 mL solution of chloroacetyl chloride (0.69 mmol, 1.1 eq) in dry dichloromethane is dropwise added to a 5 mL solution of amine (0.63 mmol, 1 eq) in dry dichloromethane at room temperature. After 4 hours of stirring, the reaction mixture is filtered over a fritted Buchner funnel to obtain solid product.

**Preparation of *alpha*-Chloro Amides: General Procedure B:** A 5 mL solution of chloroacetyl chloride (0.69 mmol, 1.1 eq) in dry dichloromethane is dropwise added to a 5 mL solution of amine (0.63 mmol, 1 eq) and 250 uL triethylamine in dry dichloromethane at room temperature. After 4 hours of stirring, the reaction mixture is filtered over a fritted Buchner funnel to obtain solid product.

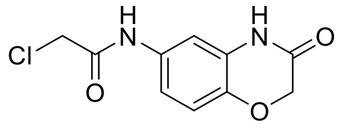

**1**

**Preparation of *2-chloro-N-(3-oxo-3,4-dihydro-2H-benzo[b][1,4]oxazin-6-yl)acetamide (1).*** Prepared according to General Procedure A on a 0.605 mmol scale to obtain 78.72 mg (0.327 mmol) of a white solid in 54% yield. A mixture of amide rotamers was observed in solution during NMR acquisition. $^{1}$H NMR (400 MHz, DMSO, 25°C): δ 10.98 (s, 1H), 10.78 (s, 1H), 10.41 (s, 1H), 7.35 (d, $J$ = 2.4 Hz, 1H), 7.08 (dd, $J$ = 8.7, 2.5 Hz, 1H), 7.03 (d, $J$ = 8.5 Hz, 1H), 6.97 (d, $J$ = 2.4 Hz, 1H), 6.94 – 6.88 (m, 2H), 4.60 (s, 2H), 4.53 (s, 2H), 4.24 (s, 2H). $^{13}$C NMR (101 MHz, DMSO, 25 °C) δ 165.49, 165.20, 164.77, 142.93, 139.88, 133.71, 128.64, 127.75, 126.72, 117.72, 117.38, 116.58, 114.46, 110.95, 107.84, 67.25, 67.16, 43.97. High-Resolution MS (ESI-): Found: 239.0232, Calc: 239.0229 for [C10H8O3N2Cl]- (δ=1.28 ppm).

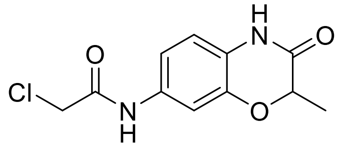

**7**

**Preparation of *2-chloro-N-(2-methyl-3-oxo-3,4-dihydro-2H-benzo[b][1,4]oxazin-7-yl)acetamide* (7)**. Prepared according to General Procedure A on a 0.552 mmol scale to obtain 118.1 mg (0.464 mmol) of a pale pink solid in 84% yield. A mixture of amide rotamers was observed in solution during NMR acquisition. $^{1}$H NMR (400 MHz, DMSO, 25°C): δ 10.83 (s, 1H), 10.64 (s, 1H), 10.43 (s, 1H), 7.34 (d, $J$ = 2.2 Hz, 1H), 7.14 (dd, $J$ = 8.5, 2.3 Hz, 1H), 6.99 – 6.94 (m, 3H), 6.84 (d, $J$ = 8.5 Hz, 1H), 4.72 (q, $J$ = 6.8 Hz, 1H), 4.64 (q, $J$ = 6.8 Hz, 1H), 4.24 (s, 2H), 1.41 (dd, $J$ = 8.0, 6.7 Hz, 7H). $^{13}$C NMR (101 MHz, DMSO, 25 °C): δ 167.00, 164.85, 143.64, 143.15, 134.41, 127.39, 124.06, 117.21, 116.61, 115.96, 113.88, 111.60, 108.37, 73.30, 73.20, 43.98, 16.56, 16.54. High-Resolution MS (ESI-): Found: 253.0389, Calc: 253.0385 for [C11H10O3N2Cl]- (δ=1.41 ppm).

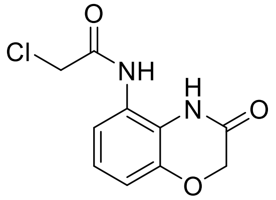

**8**

**Preparation of *2-chloro-N-(3-oxo-3,4-dihydro-2H-benzo[b][1,4]oxazin-5-yl)acetamide* (8)**. Prepared according to General Procedure A on a 0.598 mmol scale to obtain 124.35 mg (0.517 mmol) of a white solid in 86% yield. A mixture of amide rotamers was observed in solution during NMR acquisition. $^{1}$H NMR (400 MHz, DMSO, 25°C): δ 10.51 (s, 1H), 10.34 (s, 1H), 9.89 (s, 1H), 7.02 (dd, $J$ = 8.0, 1.5 Hz, 1H), 6.95 – 6.83 (m, 5H), 6.76 (dd, $J$ = 7.9, 1.6 Hz, 1H), 4.57 (d, $J$ = 6.4 Hz, 5H), 4.32 (s, 2H). $^{13}$C NMR (101 MHz, DMSO, 25 °C): δ 165.87, 165.39, 165.33, 145.22, 144.76, 125.04, 123.73, 122.82, 122.73, 120.35, 114.25, 67.16, 67.10, 43.88. High-Resolution MS (ESI-): Found: 239.0231, Calc: 239.0229 for [C10H8O3N2Cl]- (δ=0.86 ppm).

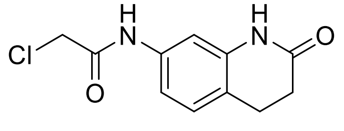

**9**

**Preparation of *2-chloro-N-(2-oxo-1,2,3,4-tetrahydroquinolin-7-yl)acetamide* (9)**. Prepared according to General Procedure A on a 0.605 mmol scale to obtain 124.44 mg (0.521 mmol) of a off-white solid in 86% yield. A mixture of amide rotamers was observed in solution during NMR acquisition. $^1$H NMR (400 MHz, DMSO, 25°C): δ 10.40 (s, 1H), 10.33 (s, 1H), 10.15 (s, 1H), 7.26 – 7.21 (m, 2H), 7.14 – 7.07 (m, 2H), 6.91 – 6.85 (m, 2H), 4.25 (s, 2H), 2.87 (dd, *J* = 8.5, 6.5 Hz, 2H), 2.81 (dd, *J* = 8.5, 6.5 Hz, 2H), 2.48 – 2.39 (m, 4H). $^{13}$C NMR (101 MHz, DMSO, 25 °C): δ 170.75, 170.68, 164.92, 139.83, 139.02, 137.92, 129.24, 128.35, 119.51, 116.38, 113.44, 109.76, 106.82, 44.02, 31.02, 30.61, 24.78. High-Resolution MS (ESI-): Found: 237.0439, Calc: 237.0436 for [C11H10O2N2Cl]- (δ=1.14 ppm).

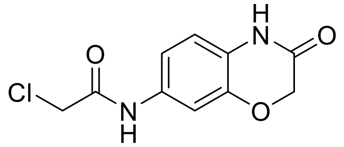

**10**

**Preparation of *2-chloro-N-(3-oxo-3,4-dihydro-2H-benzo[b][1,4]oxazin-7-yl)acetamide* (10)**. Prepared according to General Procedure B on a 0.583 mmol scale to obtain 99.47 mg (0.413 mmol) of a tan solid in 71% yield. $^1$H NMR (400 MHz, DMSO, 25°C): δ 10.68 (s, 1H), 10.26 (s, 1H), 7.30 (d, *J* = 2.2 Hz, 1H), 7.11 (dd, *J* = 8.5, 2.2 Hz, 1H), 6.84 (d, *J* = 8.5 Hz, 1H), 4.56 (s, 2H), 4.22 (s, 2H). $^{13}$C NMR (101 MHz, $CDCl_3$, 25 °C): δ 164.91, 164.84, 143.58, 134.33, 123.69, 116.22, 113.85, 108.16, 67.22, 43.99. High-Resolution MS (ESI-): Found: 239.0232, Calc: 239.0229 for [C10H8O3N2Cl]- (δ=1.28 ppm).

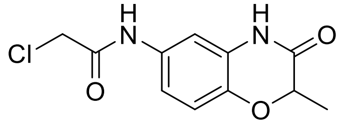

**11**

**Preparation of *2-chloro-N-(2-methyl-3-oxo-3,4-dihydro-2H-benzo[b][1,4]oxazin-6-yl)acetamide* (11)**. Prepared according to General Procedure A on a 0.533 mmol scale to obtain 92.30 mg (0.362 mmol) of a grey solid in 68% yield. A mixture of amide rotamers was observed in solution during NMR acquisition. $^1$H NMR (400 MHz, DMSO, 25°C): δ 10.93 (s, 1H), 10.72 (s, 1H), 10.41 (s, 1H), 7.35 (d, *J* = 2.4 Hz, 1H), 7.10 – 7.03 (m, 2H), 6.98 – 6.89 (m, 3H), 4.70 (d, *J* = 6.8 Hz, 1H), 4.61 (d, *J* = 6.8 Hz, 1H), 4.24 (s, 2H), 2.51 (p, *J* = 1.8 Hz, 2H), 1.41 (dd, *J* = 9.1, 6.8 Hz, 6H). $^{13}$C NMR (101 MHz, $CDCl_3$, 25 °C): δ 167.58, 167.30, 164.77, 142.62, 139.51, 133.75, 129.01, 128.12, 126.76, 117.77, 117.66, 116.84, 114.46, 110.74, 107.55, 73.16, 73.08, 43.97, 16.49, 16.44. High-Resolution MS (ESI-): Found: 253.0389, Calc: 253.0385 for [C11H10O3N2Cl]- (δ=1.41 ppm).

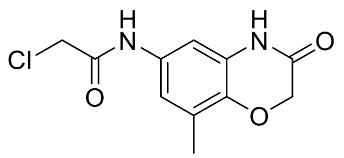

**12**

**Preparation of *2-chloro-N-(8-methyl-3-oxo-3,4-dihydro-2H-benzo[b][1,4]oxazin-6-yl)acetamide* (12)**. Prepared according to General Procedure B on a 0.535 mmol scale to obtain 100.2 mg (0.393 mmol) of a light brown solid in 74% yield. A mixture of amide rotamers was observed in solution during NMR acquisition. $^{1}$H NMR (400 MHz, DMSO, 25°C): δ 10.90 (s, 1H), 10.71 (s, 3H), 10.28 (s, 3H), 7.17 (d, *J* = 2.4 Hz, 3H), 6.98 (dd, *J* = 2.4, 0.8 Hz, 3H), 6.82 – 6.77 (m, 2H), 4.61 (s, 2H), 4.54 (s, 7H), 4.22 (s, 7H), 2.51 (d, *J* = 1.9 Hz, 4H), 2.17 (s, 3H), 2.13 (s, 10H). $^{13}$C NMR (101 MHz, $CDCl_3$, 25 °C): δ 165.60, 165.33, 164.72, 138.14, 133.06, 128.21, 127.40, 127.04, 125.76, 118.87, 115.97, 108.67, 105.76, 67.30, 43.98, 15.84. High-Resolution MS (ESI-): Found: 253.0389, Calc: 253.0385 for [C11H10O3N2Cl]- (δ=1.41 ppm).

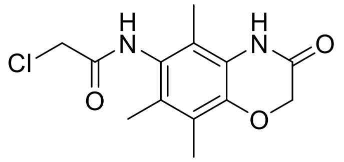

**13**

**Preparation of *2-chloro-N-(5,7,8-trimethyl-3-oxo-3,4-dihydro-2H-benzo[b][1,4]oxazin-6-yl)acetamide* (13)**. Prepared according to General Procedure A on a 0.478 mmol scale to obtain 110.8 mg (0.392 mmol) of an off-white solid in 82% yield. A mixture of amide rotamers was observed in solution during NMR acquisition. $^{1}$H NMR (400 MHz, DMSO, 25°C): δ 10.30 (s, 1H), 10.14 (s, 1H), 9.67 (s, 1H), 4.53 – 4.48 (m, 5H), 4.28 (s, 3H), 2.53 – 2.49 (m, 3H), 2.22 (d, *J* = 11.9 Hz, 6H), 2.11 (d, *J* = 8.2 Hz, 7H), 2.01 (d, *J* = 6.2 Hz, 8H). $^{13}$C NMR (101 MHz, $CDCl_3$, 25 °C): δ 169.87, 167.64, 165.57, 129.38, 128.94, 124.82, 124.08, 121.98, 67.37, 43.20, 14.74, 14.50, 12.57, 12.33. High-Resolution MS (ESI-): Found: 317.0469, Calc: 317.0465 for [C13H15O3N2Cl2]- (δ=1.19 ppm).

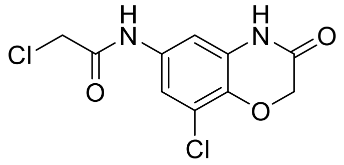

**14**

**Preparation of *2-chloro-N-(8-chloro-3-oxo-3,4-dihydro-2H-benzo[b][1,4]oxazin-6-yl)acetamide* (14)**. Prepared according to General Procedure B on a 0.464 mmol scale to obtain 51.6 mg (0.188 mmol) of an off-white solid in 40% yield. A mixture of amide rotamers was observed in solution during NMR acquisition. $^{1}$H NMR (400 MHz, DMSO, 25°C): δ 10.99 (s, 1H), 10.39 (s, 1H), 7.34 (d, *J* = 2.4 Hz, 1H), 7.18 (d, *J* = 2.4 Hz, 1H), 4.67 (s, 2H), 4.23 (s, 2H), 3.35 (s, 1H), 2.51 (d, *J* = 1.9 Hz, 2H). $^{13}$C NMR (101 MHz, $CDCl_3$, 25 °C): δ 165.16, 165.11, 136.02, 133.81, 129.17, 120.37, 114.32, 106.32, 67.46, 43.92. High-Resolution MS (ESI-): Found: 272.9843, Calc: 272.9839 for [C10H7O3N2Cl2]- (δ=1.39 ppm).

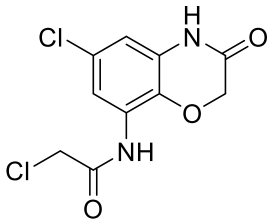

**15**

**Preparation of *2-chloro-N-(6-chloro-3-oxo-3,4-dihydro-2H-benzo[b][1,4]oxazin-8-yl)acetamide* (15)**. Prepared according to General Procedure B on a 0.468 mmol scale to obtain 47.0 mg (0.171mmol) of a maroon solid in 37% yield. $^{1}$H NMR (400 MHz, DMSO, 25°C): 10.89 (s, 1H), 9.89 (s, 1H), 7.70 (d, *J* = 2.5 Hz, 1H), 6.71 (d, *J* = 2.5 Hz, 1H), 4.67 (s, 2H), 4.39 (s, 2H), 3.35 (s, 2H), 2.53 – 2.48 (m, 2H). $^{13}$C NMR (101 MHz, $CDCl_3$, 25 °C): δ 165.80, 165.09, 133.13, 129.19, 127.99, 125.81, 115.46, 111.29, 67.39, 43.66. High-Resolution MS (ESI-): Found: 272.9842, Calc: 272.9839 for [C10H7O3N2Cl2]- (δ=1.02 ppm).

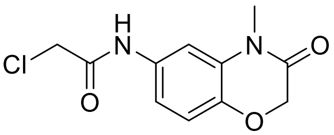

**16**

**Preparation of *2-chloro-N-(4-methyl-3-oxo-3,4-dihydro-2H-benzo[b][1,4]oxazin-6-yl)acetamide* (16)**. Prepared according to General Procedure B on a 0.533 mmol scale to obtain 104.4 mg (0.409 mmol) of a dark brown solid in 77% yield. $^{1}$H NMR (400 MHz, DMSO, 25°C): δ 10.33 (s, 1H), 7.45 (d, *J* = 2.4 Hz, 1H), 7.20 (dd, *J* = 8.6, 2.3 Hz, 1H), 6.98 (d, *J* = 8.6 Hz, 1H), 4.63 (s, 2H), 4.25 (s, 2H), 3.25 (s, 4H). $^{13}$C NMR (101 MHz, $CDCl_3$, 25 °C): δ 164.92, 164.67, 141.28, 134.03, 129.94, 116.73, 114.78, 107.33, 67.53, 43.98, 28.08. High-Resolution MS (ESI-): Found: 253.0388, Calc: 253.0385 for [C11H10O3N2Cl]- (δ=1.01 ppm).

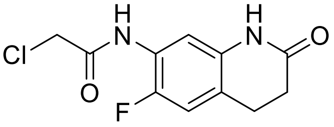

**17**

**Preparation of *2-chloro-N-(6-fluoro-2-oxo-1,2,3,4-tetrahydroquinolin-7-yl)acetamide* (17)**. Prepared according to General Procedure B on a 0.551 mmol scale to obtain 86.70 mg (0.338 mmol) of a light tan solid in 61% yield. $^{1}$H NMR (400 MHz, DMSO, 25°C): δ 10.09 (d, *J* = 33.5 Hz, 2H), 7.44 (s, 1H), 7.14 (d, *J* = 11.2 Hz, 1H), 4.34 (s, 2H), 2.89 – 2.77 (m, 2H), 2.47 (d, *J* = 33.2 Hz, 3H). $^{13}$C NMR (101 MHz, $CDCl_3$, 25 °C): δ 170.39, 165.46, 134.95, 124.24, 124.11, 121.63, 115.20, 114.99, 110.90, 43.54, 30.50, 24.79. High-Resolution MS (ESI-): Found: 255.0348, Calc: 255.00342 for [C11H9O2N2ClF]- (δ=2.33 ppm).

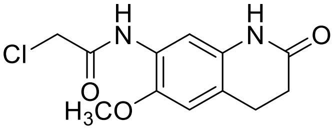

**18**

**Preparation of *2-chloro-N-(6-methoxy-2-oxo-1,2,3,4-tetrahydroquinolin-7-yl)acetamide* (18)**. Prepared according to General Procedure B on a 0.256 mmol scale to obtain 48.69 mg (0.181 mmol) of a dark brown solid in 71% yield. $^{1}H$ NMR (400 MHz, DMSO, 25°C): δ 9.94 (s, 1H), 7.61 (s, 1H), 6.93 (s, 1H), 4.37 (s, 2H), 3.80 (s, 3H), 2.83 (dd, $J$ = 8.6, 6.5 Hz, 2H), 2.41 (dd, $J$ = 8.5, 6.5 Hz, 2H). $^{13}C$ NMR (101 MHz, $CDCl_3$, 25 °C): δ 170.26, 164.99, 145.14, 131.73, 125.53, 120.24, 111.60, 109.55, 56.60, 43.81, 30.93, 25.34. High-Resolution MS (ESI-): Found: 267.0542, Calc: 267.0546 for [C12H12O3N2Cl]- (δ=1.52 ppm).

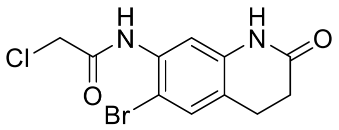

**20**

**Preparation of *N-(6-bromo-2-oxo-1,2,3,4-tetrahydroquinolin-7-yl)-2-chloroacetamide* (20)**. Prepared according to General Procedure B on a 0.42 mmol scale to obtain 98.9 mg (0.31 mmol) of a solid in 74% yield. $^{1}H$ NMR (400 MHz, DMSO, 25°C): δ 10.22 (s, 1H), 9.74 (s, 1H), 7.48 (s, 1H), 7.21 (s, 1H), 4.34 (s, 2H), 2.87 (t, J = 7.5 Hz, 2H), 2.44 (t, J = 7.6 Hz, 2H). $^{13}C$ NMR (100 MHz, $CDCl_3$, 25 °C): δ 170.02, 165.00, 138.19, 133.98, 131.22, 123.40, 112.97, 110.70, 42.99, 29.99, 24.02. High-Resolution MS (ESI-): Found: 314.9546, Calc: 314.9541 for [C11H9O2N2BrCl]- (δ=1.46 ppm).

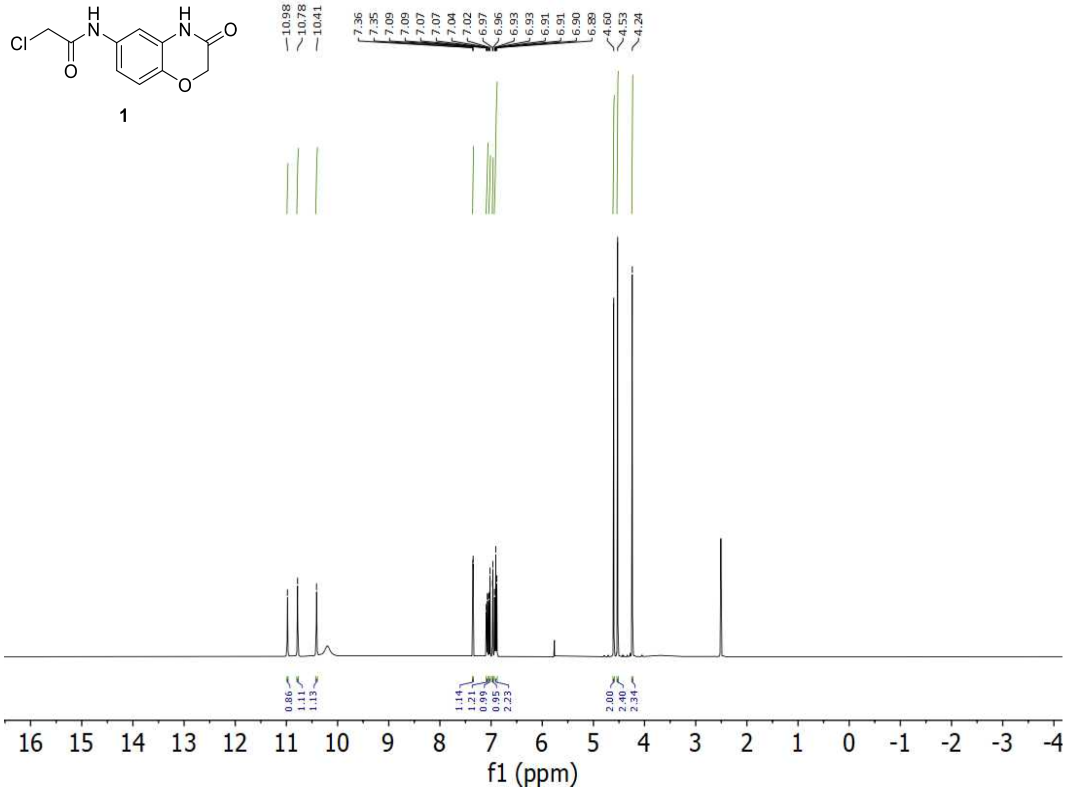

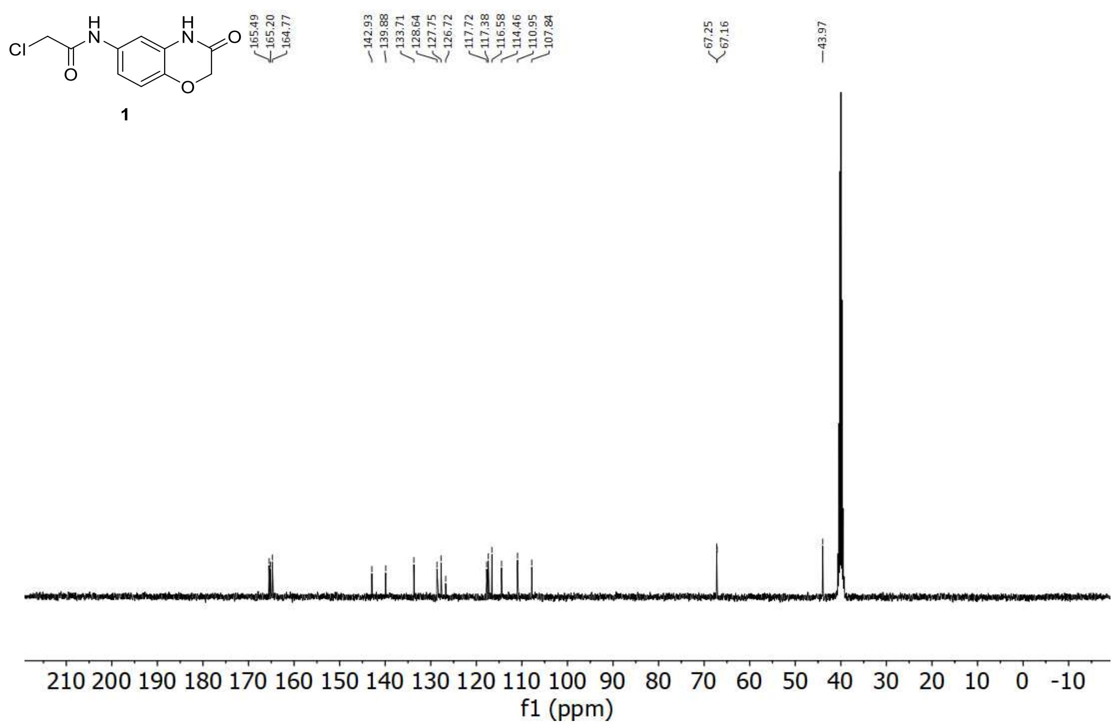
Cl
N
H
H
N
O
O
O
1
165.49
165.20
164.77
142.93
139.88
133.71
128.64
127.75
126.72
117.72
117.38
116.58
114.46
110.95
107.84
67.25
67.16
43.97
210 200 190 180 170 160 150 140 130 120 110 100 90 80 70 60 50 40 30 20 10 0 -10
f1 (ppm)

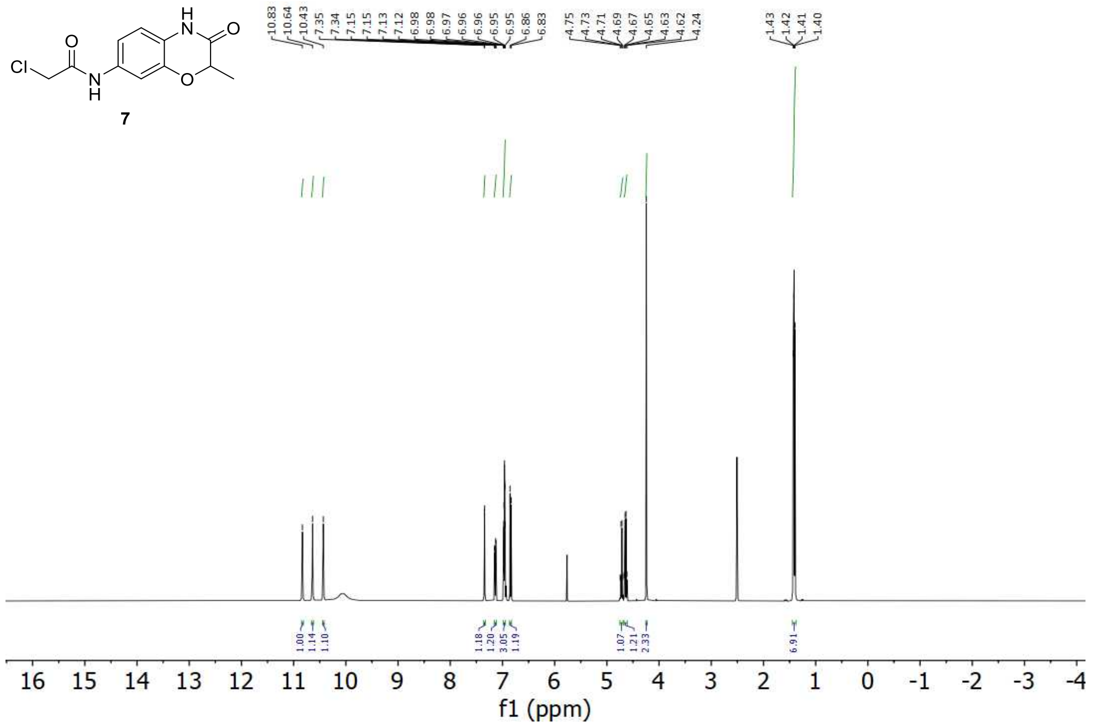
Cl
N
H
H
N
O
O
O
7
10.83
10.64
10.43
7.35
7.34
7.15
7.15
7.13
7.12
6.98
6.98
6.97
6.96
6.96
6.95
6.95
6.86
6.83
4.75
4.73
4.71
4.69
4.67
4.65
4.63
4.62
4.24
1.43
1.42
1.41
1.40
1.00
1.14
1.10
1.18
1.20
3.05
1.19
1.07
1.21
2.33
6.91
16 15 14 13 12 11 10 9 8 7 6 5 4 3 2 1 0 -1 -2 -3 -4
f1 (ppm)

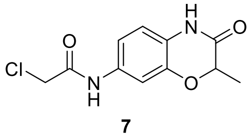

**7**

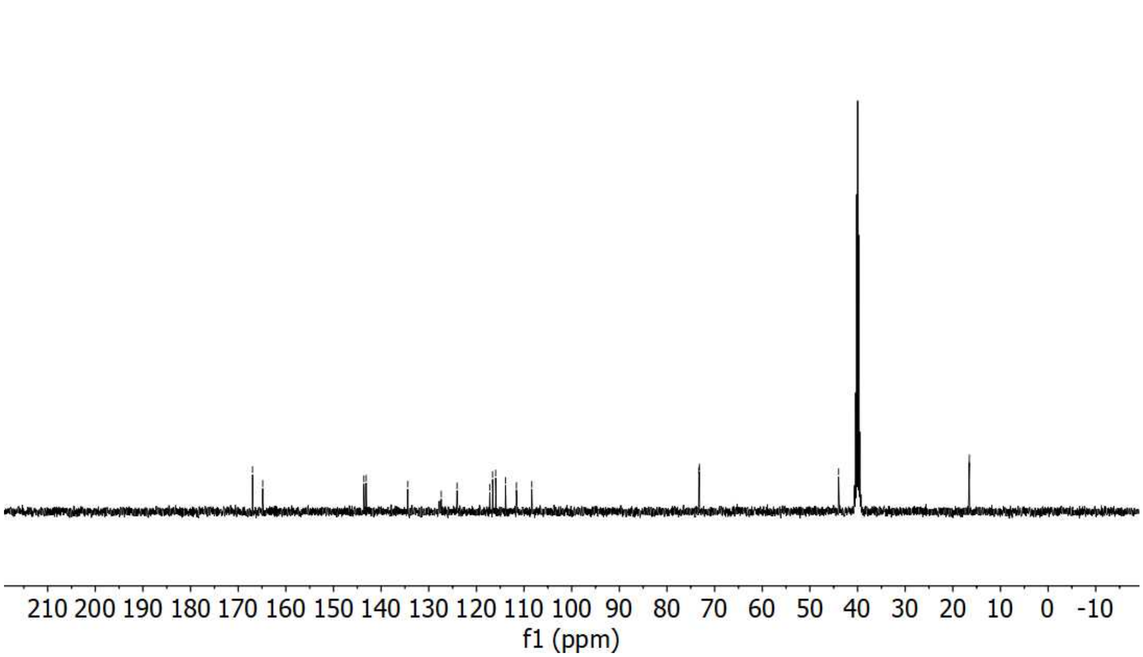
167.00
164.85
143.64
143.15
134.41
127.39
124.06
117.21
116.61
115.96
113.88
111.60
108.37
73.30
73.20
43.98
16.56
16.54
210 200 190 180 170 160 150 140 130 120 110 100 90 80 70 60 50 40 30 20 10 0 -10
f1 (ppm)

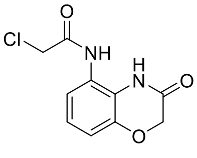

**8**

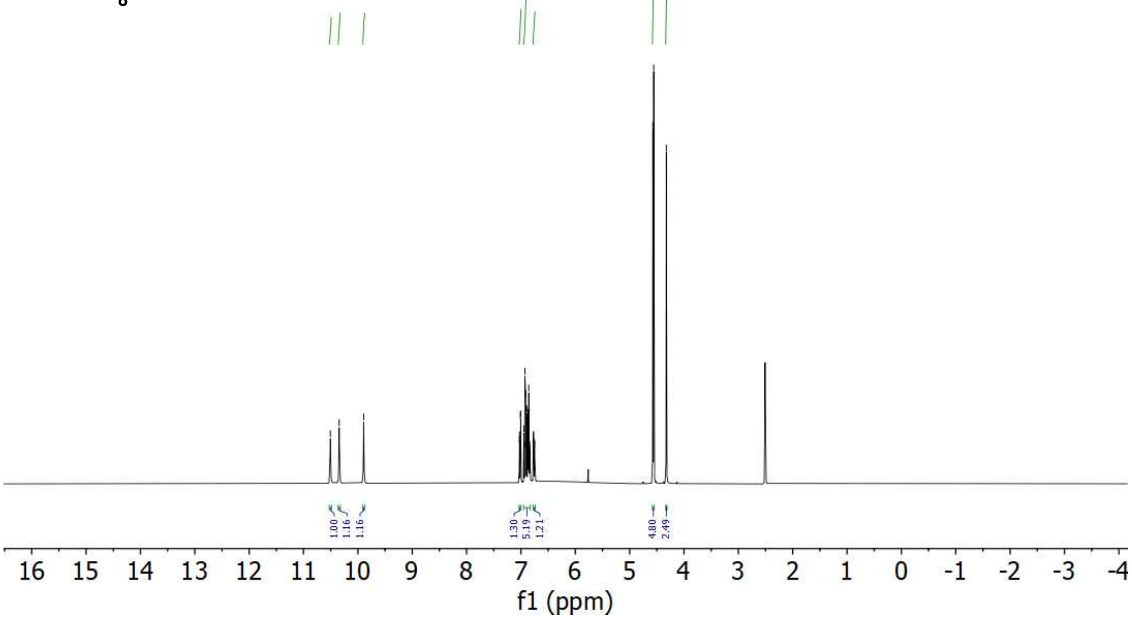
10.51
10.34
9.89
7.03
7.03
7.01
7.01
6.95
6.93
6.93
6.91
6.91
6.89
6.88
6.87
6.86
6.86
6.85
6.84
6.84
6.77
6.77
6.75
6.75
4.57
4.56
4.32
1.00
1.16
1.16
1.30
5.19
1.21
4.80
2.49
16 15 14 13 12 11 10 9 8 7 6 5 4 3 2 1 0 -1 -2 -3 -4
f1 (ppm)

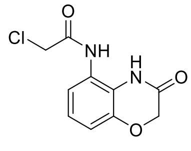

**8**

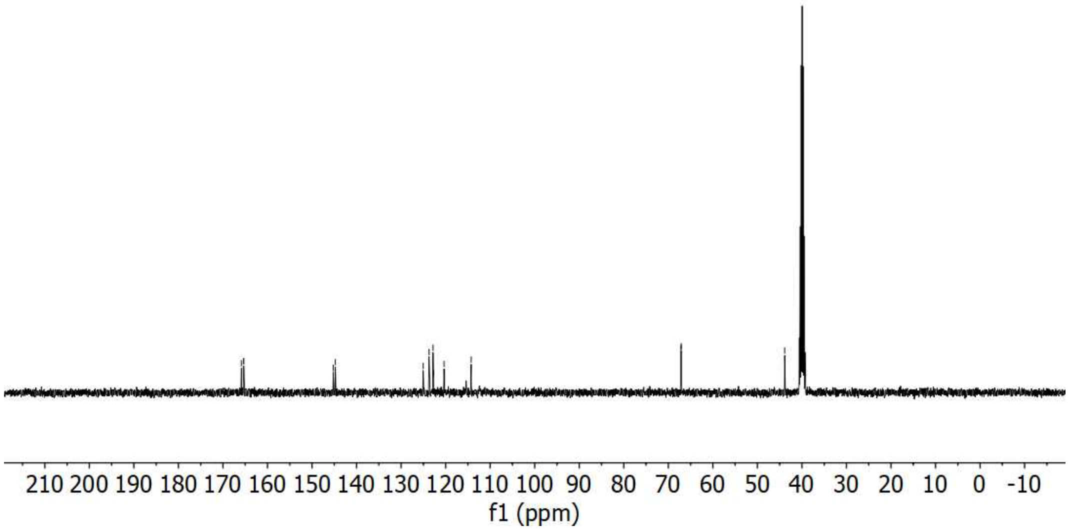
165.87
165.39
165.33
145.22
144.76
125.04
123.73
122.82
122.73
120.35
114.25
67.16
67.10
43.88
210 200 190 180 170 160 150 140 130 120 110 100 90 80 70 60 50 40 30 20 10 0 -10
f1 (ppm)

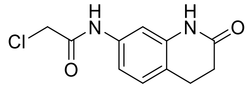

**9**

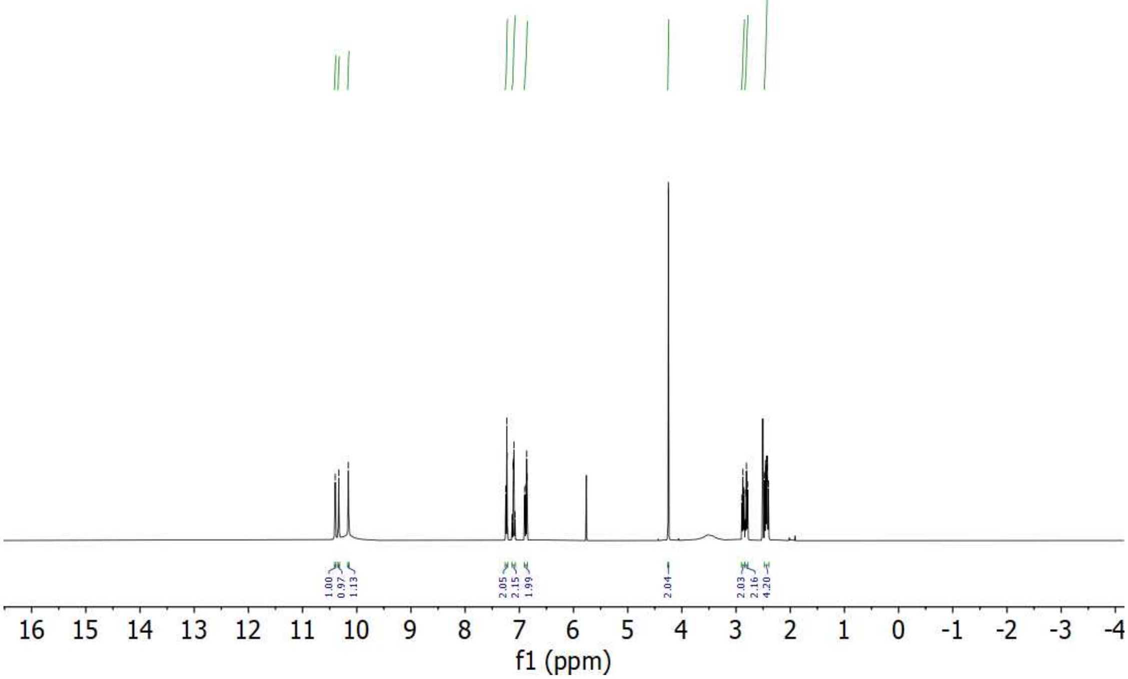
10.40
10.33
10.15
7.25
7.23
7.23
7.13
7.13
7.11
7.11
7.10
7.08
6.90
6.90
6.88
6.88
6.86
6.86
4.25
2.89
2.87
2.87
2.85
2.83
2.81
2.80
2.79
2.47
2.46
2.45
2.45
2.44
2.44
2.42
2.42
2.42
2.40
1.00
0.97
1.13
2.05
2.15
1.99
2.04
2.03
2.16
4.20
16 15 14 13 12 11 10 9 8 7 6 5 4 3 2 1 0 -1 -2 -3 -4
f1 (ppm)

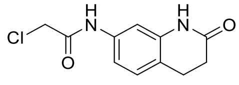
Cl
H
N
H
N
O
O

**9**

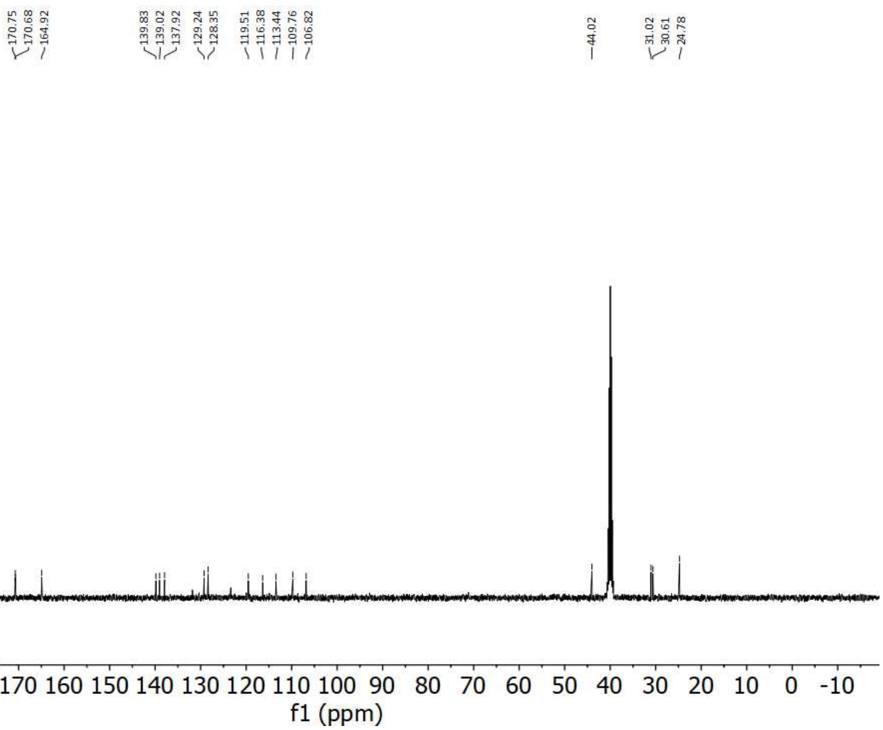
170.75
170.68
164.92
139.83
139.02
137.92
129.24
128.35
119.51
116.38
113.44
109.76
106.82
44.02
31.02
30.61
24.78
210 200 190 180 170 160 150 140 130 120 110 100 90 80 70 60 50 40 30 20 10 0 -10
f1 (ppm)

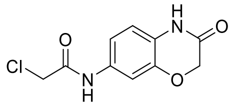
H
N
O
O
Cl
N
H
O

**10**

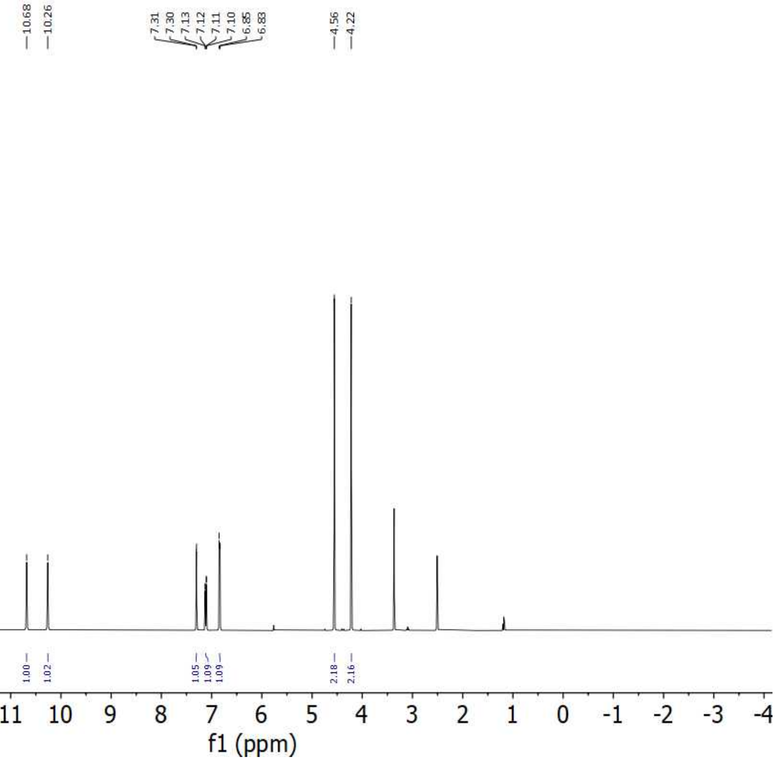
10.68
10.26
7.31
7.30
7.13
7.12
7.11
7.10
6.85
6.83
4.56
4.22
1.00
1.02
1.05
1.09
1.09
2.18
2.16
16 15 14 13 12 11 10 9 8 7 6 5 4 3 2 1 0 -1 -2 -3 -4
f1 (ppm)

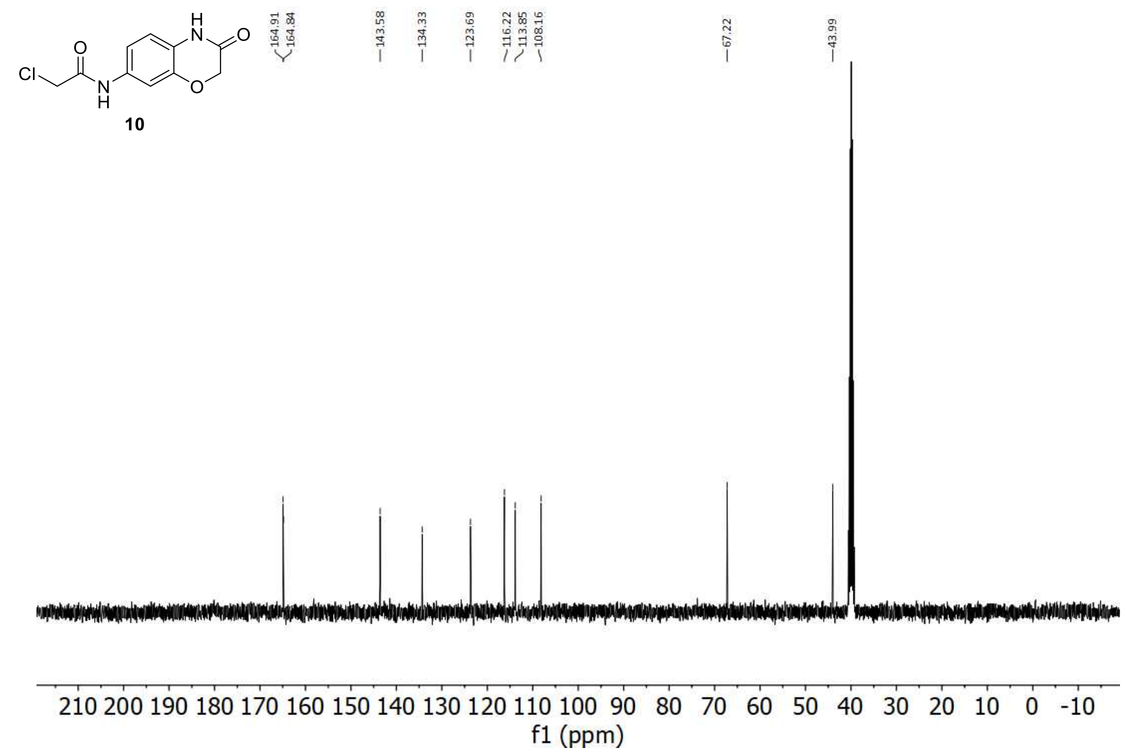
10
164.91
164.84
143.58
134.33
123.69
116.22
113.85
108.16
67.22
43.99
210 200 190 180 170 160 150 140 130 120 110 100 90 80 70 60 50 40 30 20 10 0 -10
f1 (ppm)

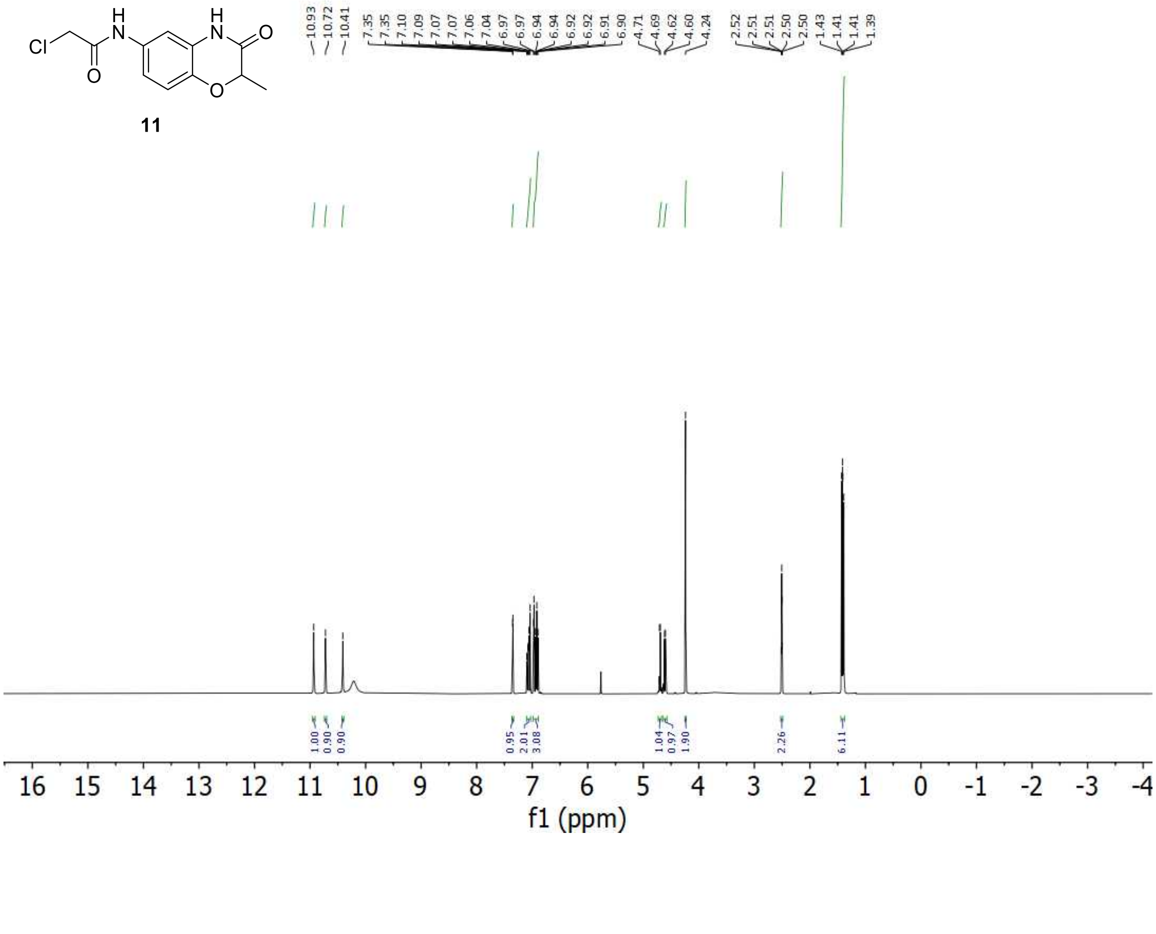
11
10.93
10.72
10.41
7.35
7.35
7.10
7.09
7.07
7.07
7.06
7.04
6.97
6.97
6.94
6.94
6.92
6.92
6.91
6.90
4.71
4.69
4.62
4.60
4.24
2.52
2.51
2.51
2.50
2.50
1.43
1.41
1.41
1.39
1.00
0.90
0.90
0.95
2.01
3.08
1.04
0.97
1.90
2.26
6.11
16 15 14 13 12 11 10 9 8 7 6 5 4 3 2 1 0 -1 -2 -3 -4
f1 (ppm)

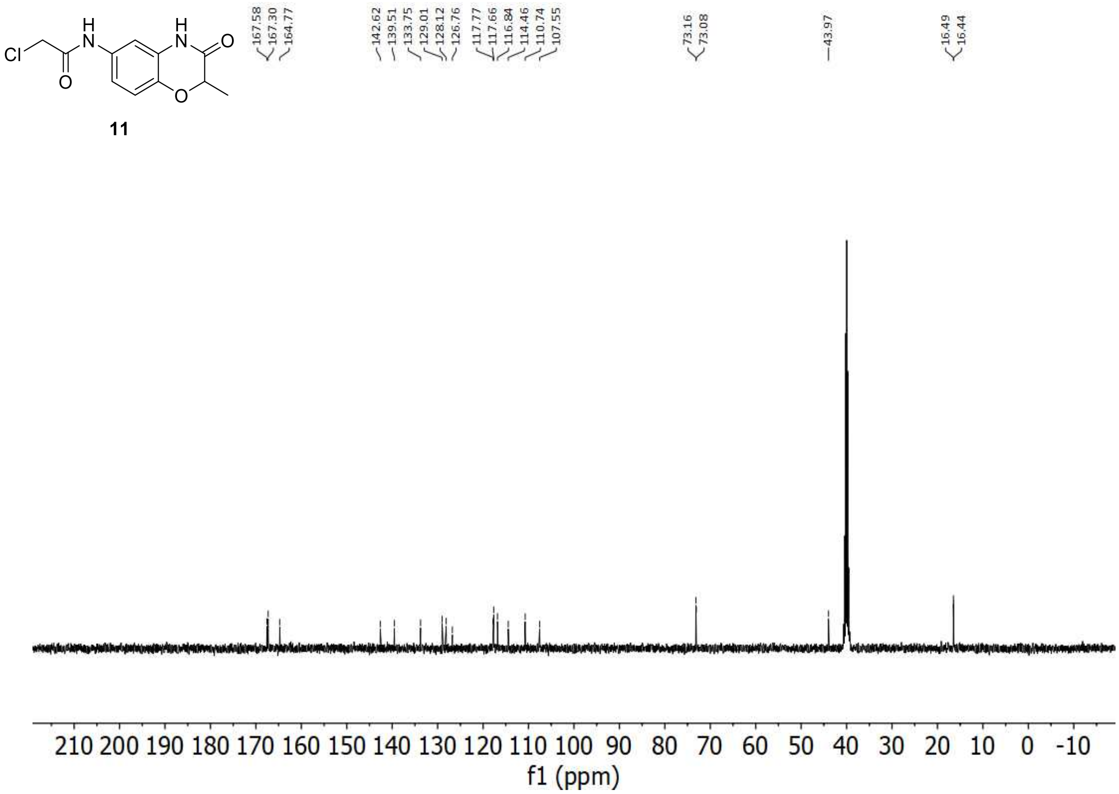
167.58
167.30
164.77
142.62
139.51
133.75
129.01
128.12
126.76
117.77
117.66
116.84
114.46
110.74
107.55
73.16
73.08
43.97
16.49
16.44
210 200 190 180 170 160 150 140 130 120 110 100 90 80 70 60 50 40 30 20 10 0 -10
f1 (ppm)

11

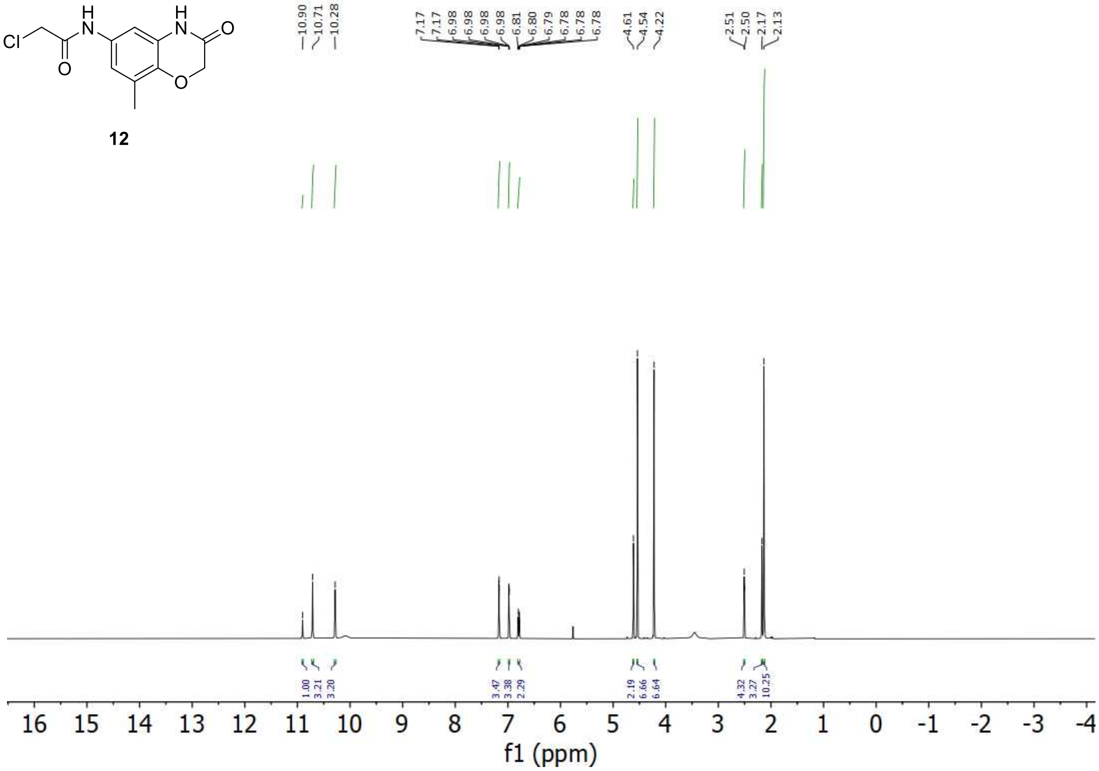
10.90
10.71
10.28
7.17
7.17
6.98
6.98
6.98
6.98
6.81
6.80
6.79
6.78
6.78
6.78
4.61
4.54
4.22
2.51
2.50
2.17
2.13
1.00
3.21
3.20
3.47
3.38
2.29
2.19
6.66
6.64
4.32
3.27
10.25
16 15 14 13 12 11 10 9 8 7 6 5 4 3 2 1 0 -1 -2 -3 -4
f1 (ppm)

12

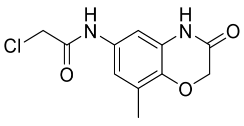

**12**

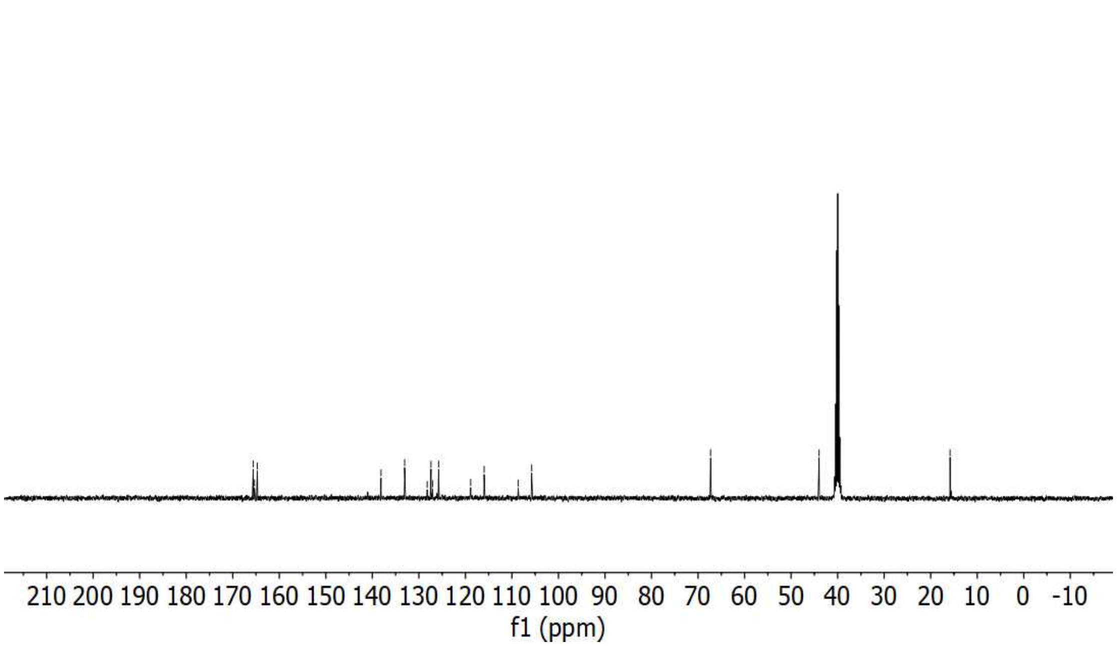
165.60
165.33
164.72
138.14
133.06
128.21
127.40
127.04
125.76
118.87
115.97
108.67
105.76
67.30
43.98
15.84
210 200 190 180 170 160 150 140 130 120 110 100 90 80 70 60 50 40 30 20 10 0 -10
f1 (ppm)

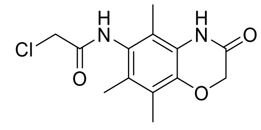

**13**

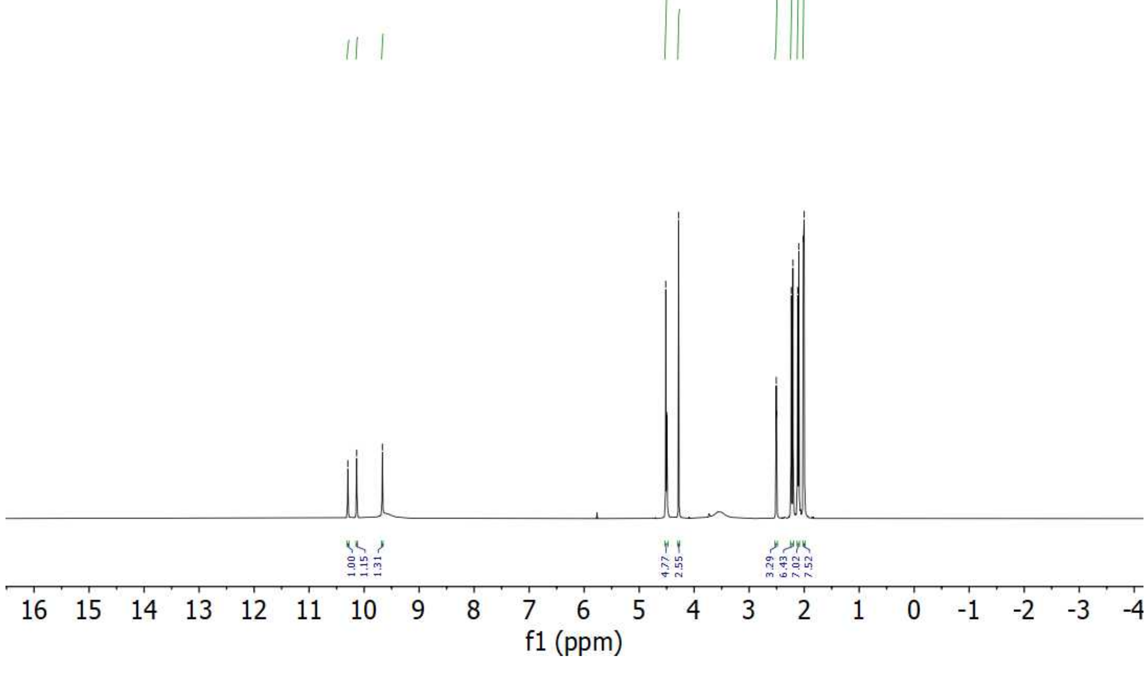
10.30
10.14
9.67
4.52
4.50
4.50
4.28
2.51
2.51
2.50
2.23
2.21
2.12
2.10
2.02
2.00
1.00
1.15
1.31
4.77
2.55
3.29
6.43
7.02
7.52
16 15 14 13 12 11 10 9 8 7 6 5 4 3 2 1 0 -1 -2 -3 -4
f1 (ppm)

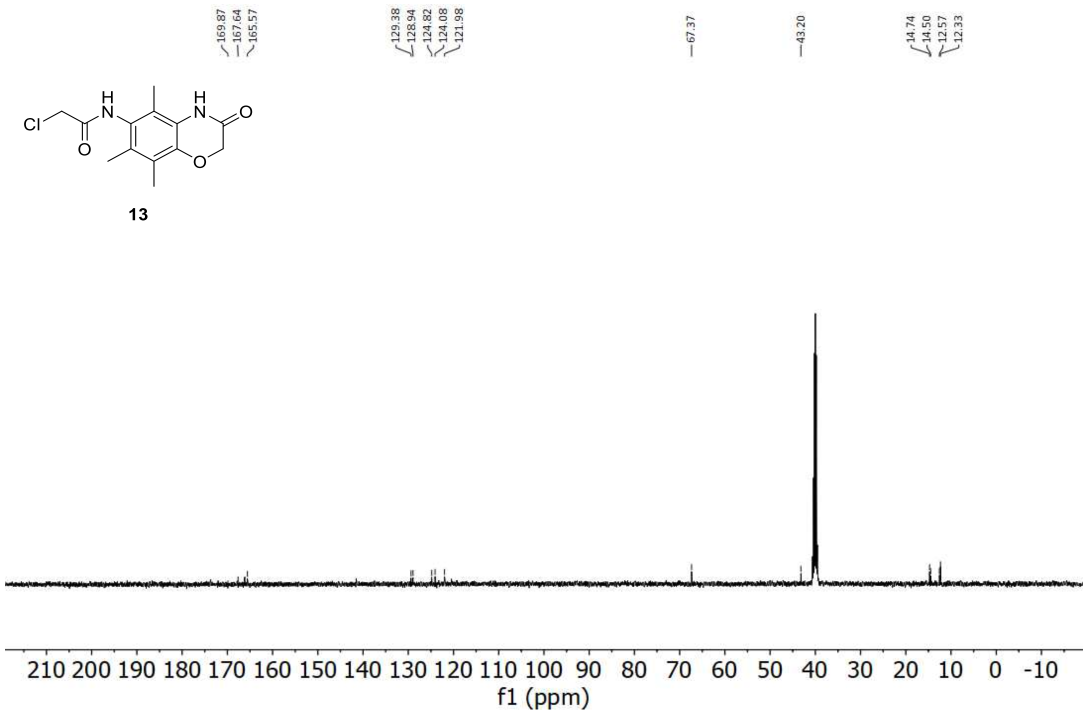
169.87
167.64
165.57
129.38
128.94
124.82
124.08
121.98
67.37
43.20
14.74
14.50
12.57
12.33
Cl
H
N
O
13
210 200 190 180 170 160 150 140 130 120 110 100 90 80 70 60 50 40 30 20 10 0 -10
f1 (ppm)

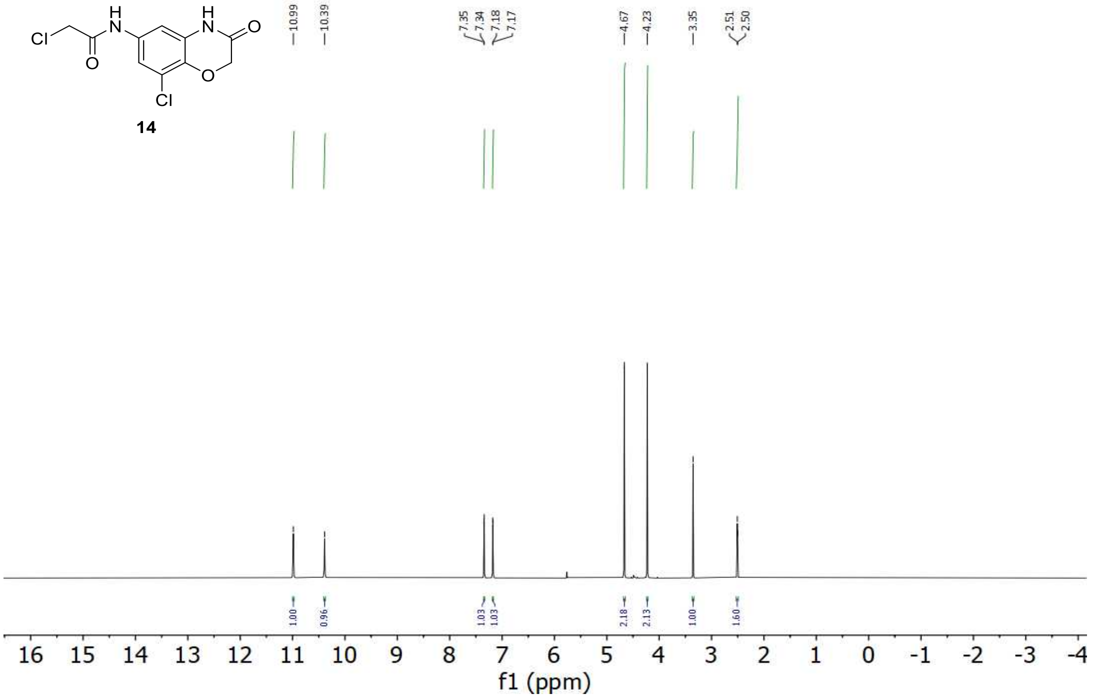
Cl
H
N
O
14
10.99
10.39
7.35
7.34
7.18
7.17
4.67
4.23
3.35
2.51
2.50
1.00
0.96
1.03
1.03
2.18
2.13
1.00
1.60
16 15 14 13 12 11 10 9 8 7 6 5 4 3 2 1 0 -1 -2 -3 -4
f1 (ppm)

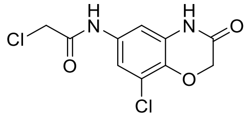
Cl
H
N
O
Cl

**14**

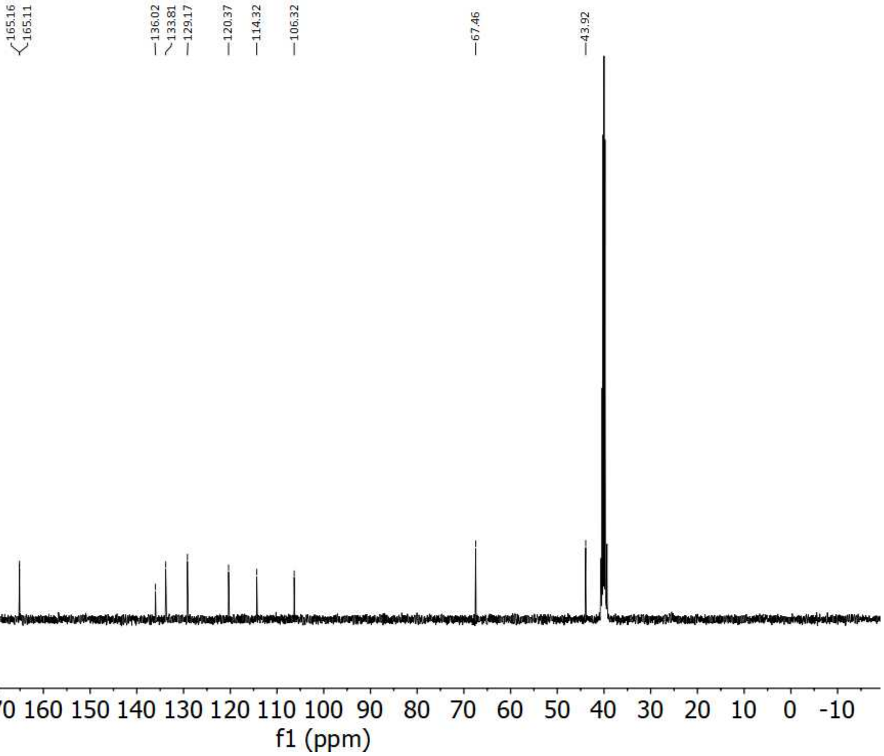
165.16
165.11
136.02
133.81
129.17
120.37
114.32
106.32
67.46
43.92
210 200 190 180 170 160 150 140 130 120 110 100 90 80 70 60 50 40 30 20 10 0 -10
f1 (ppm)

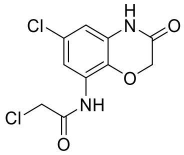
Cl
H
N
O
NH
Cl
O

**15**

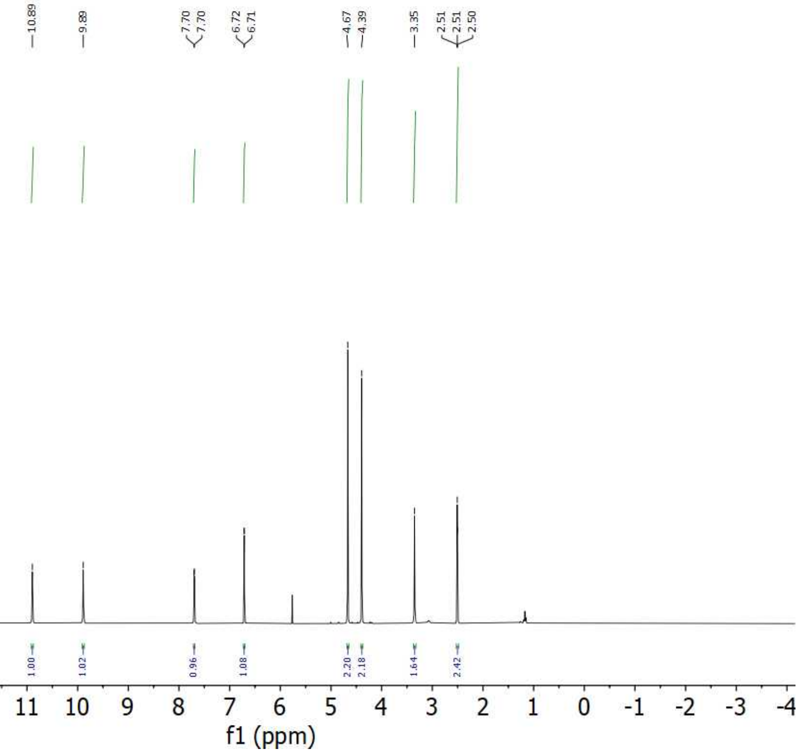
10.89
9.89
7.70
7.70
6.72
6.71
4.67
4.39
3.35
2.51
2.51
2.50
1.00
1.02
0.96
1.08
2.20
2.18
1.64
2.42
16 15 14 13 12 11 10 9 8 7 6 5 4 3 2 1 0 -1 -2 -3 -4
f1 (ppm)

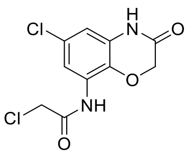

Cl
H
N
O
O
NH
Cl
O


**15**

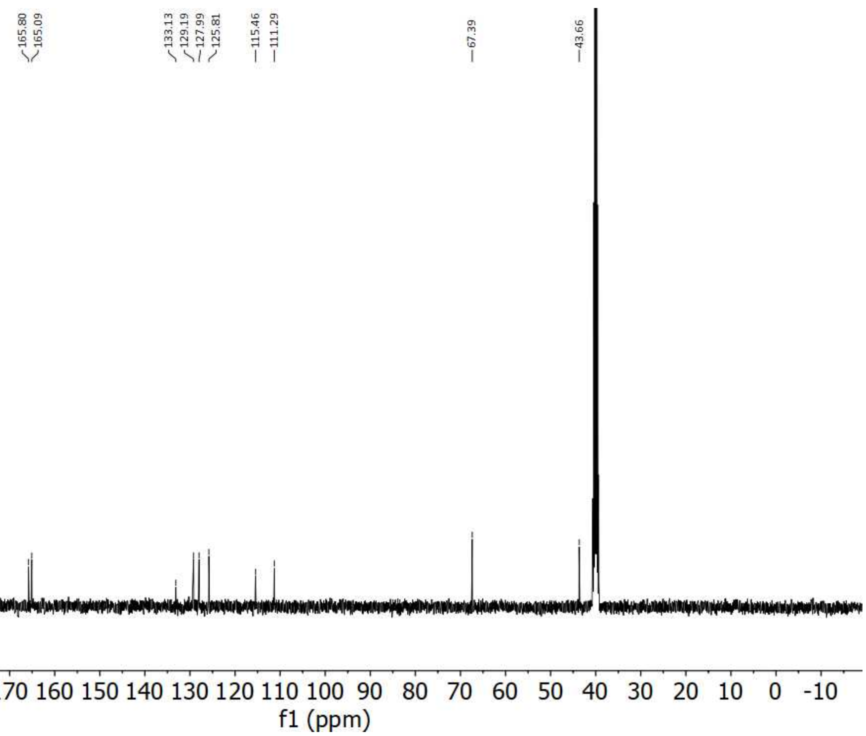

165.80
165.09
133.13
129.19
127.99
125.81
115.46
111.29
67.39
43.66
210 200 190 180 170 160 150 140 130 120 110 100 90 80 70 60 50 40 30 20 10 0 -10
f1 (ppm)


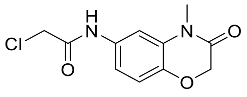

Cl
H
N
O
N
O
O


**16**

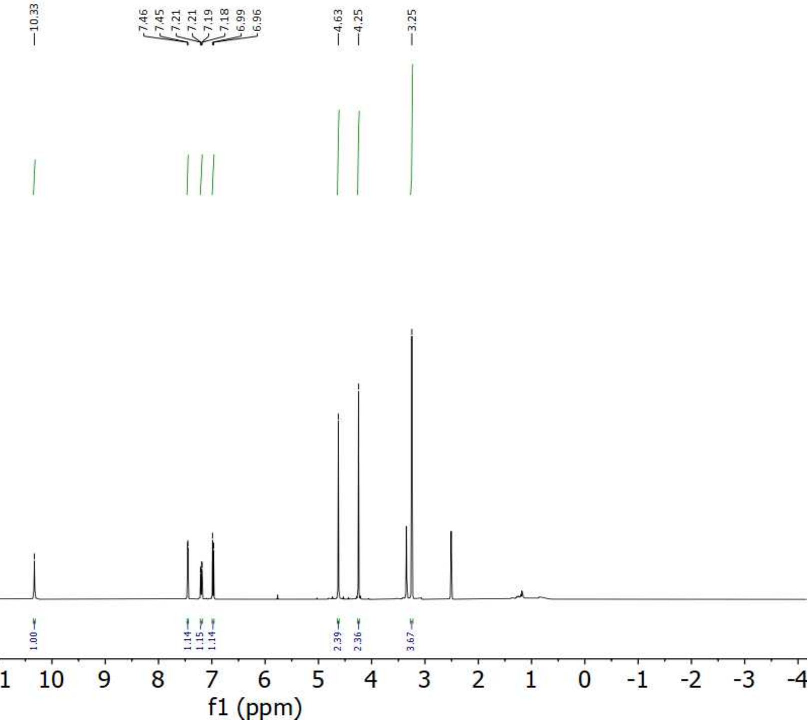

10.33
7.46
7.45
7.21
7.21
7.19
7.18
6.99
6.96
4.63
4.25
3.25
1.00
1.14
1.15
1.14
2.39
2.36
3.67
16 15 14 13 12 11 10 9 8 7 6 5 4 3 2 1 0 -1 -2 -3 -4
f1 (ppm)

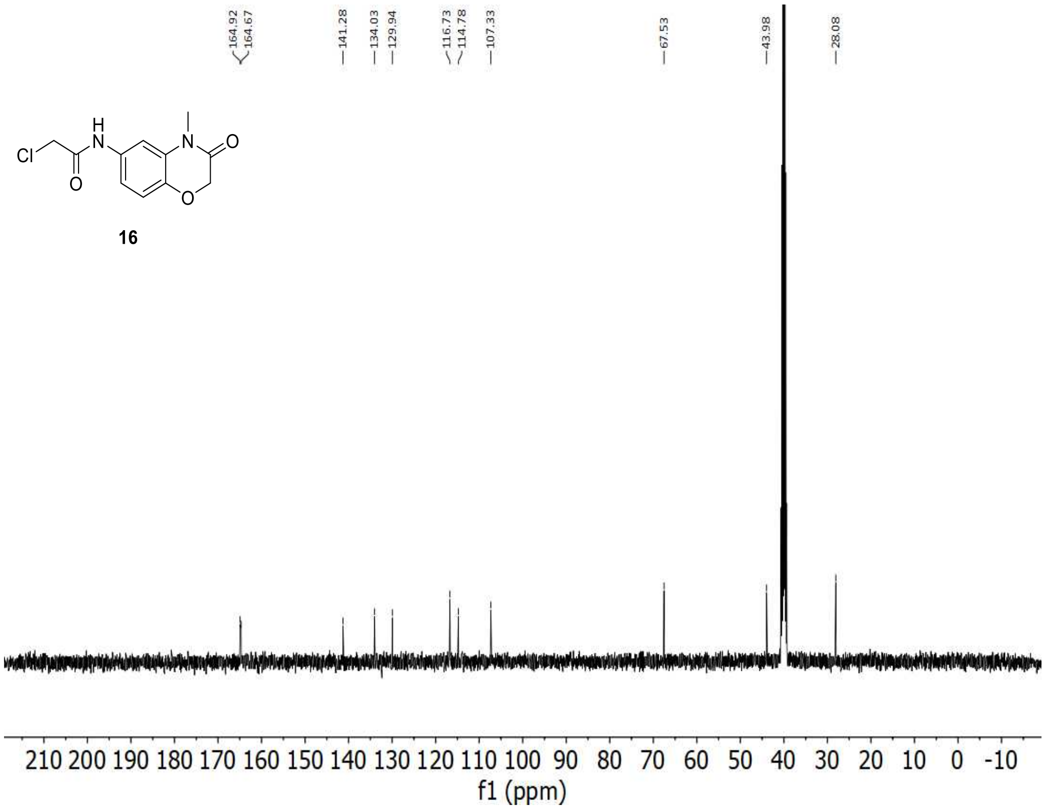
164.92
164.67
141.28
134.03
129.94
116.73
114.78
107.33
67.53
43.98
28.08
Cl
H
N
N
O
O
O
210 200 190 180 170 160 150 140 130 120 110 100 90 80 70 60 50 40 30 20 10 0 -10
f1 (ppm)

**16**

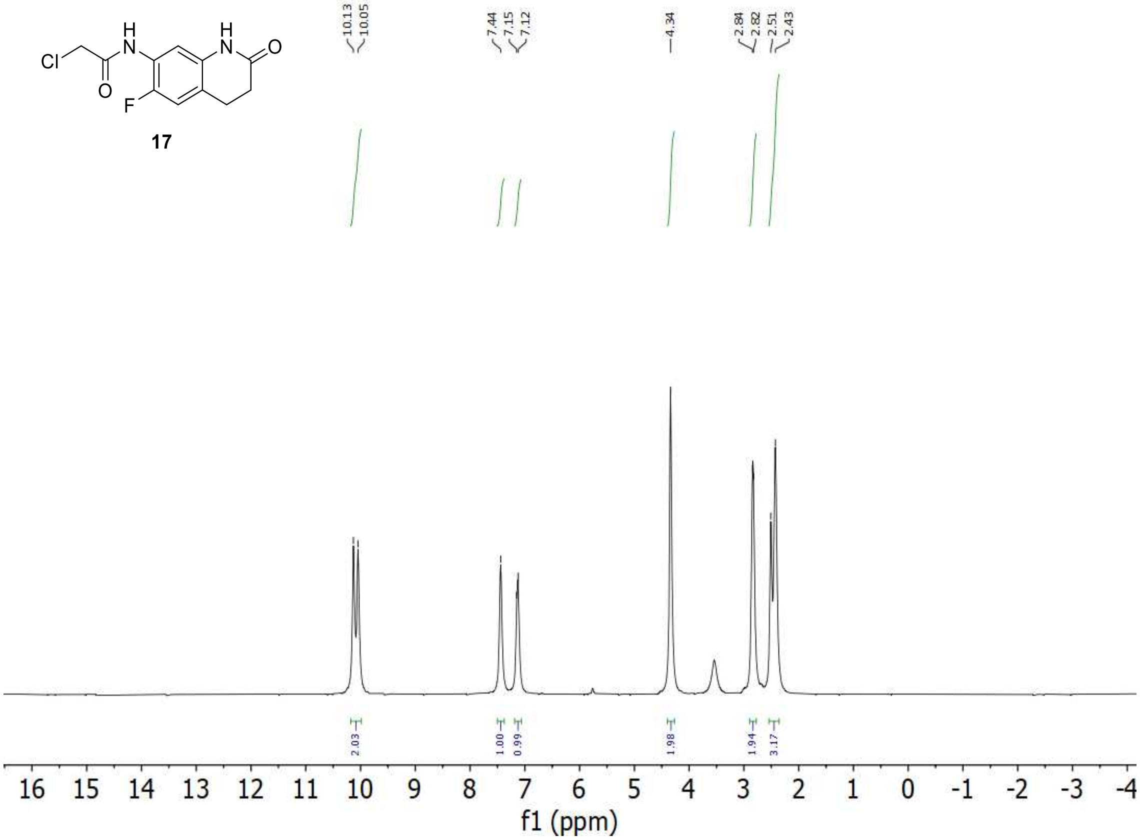
Cl
H
N
H
N
O
O
F
10.13
10.05
7.44
7.15
7.12
4.34
2.84
2.82
2.51
2.43
2.03
1.00
0.99
1.98
1.94
3.17
16 15 14 13 12 11 10 9 8 7 6 5 4 3 2 1 0 -1 -2 -3 -4
f1 (ppm)

**17**

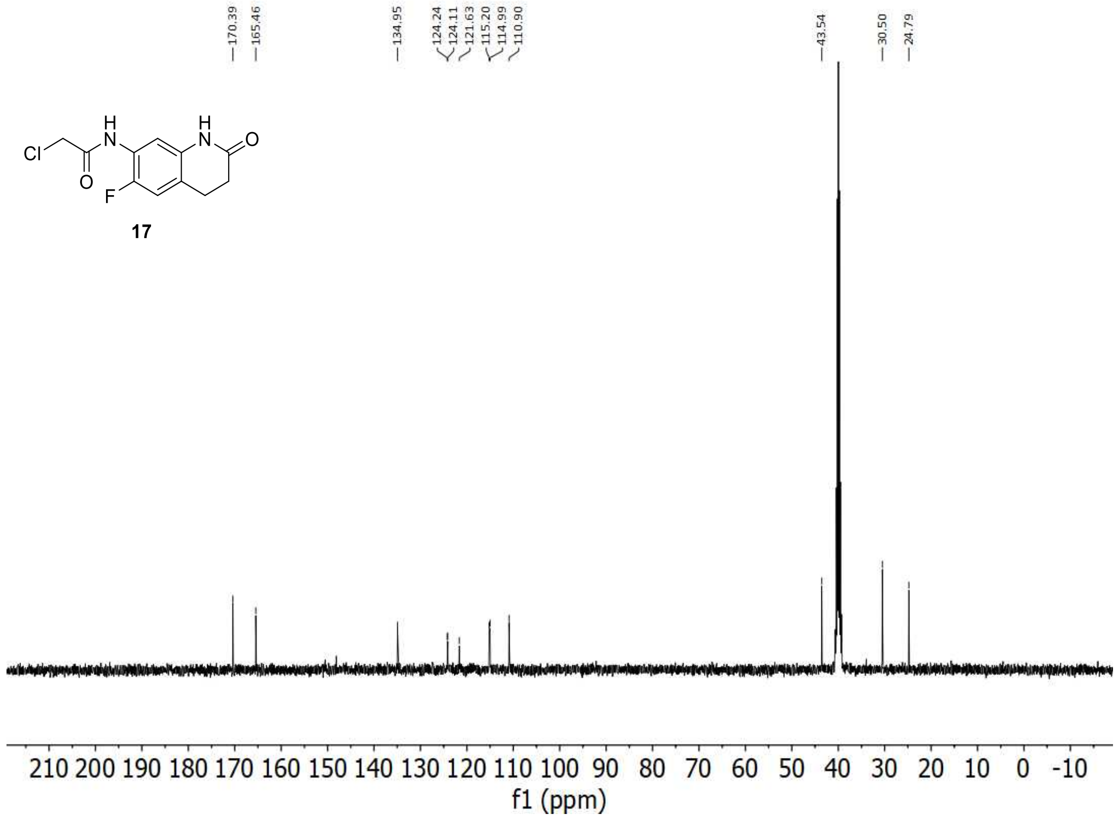
Cl
H
N
O
F
H
N
O
170.39
165.46
134.95
124.24
124.11
121.63
115.20
114.99
110.90
43.54
30.50
24.79
210 200 190 180 170 160 150 140 130 120 110 100 90 80 70 60 50 40 30 20 10 0 -10
f1 (ppm)

**17**

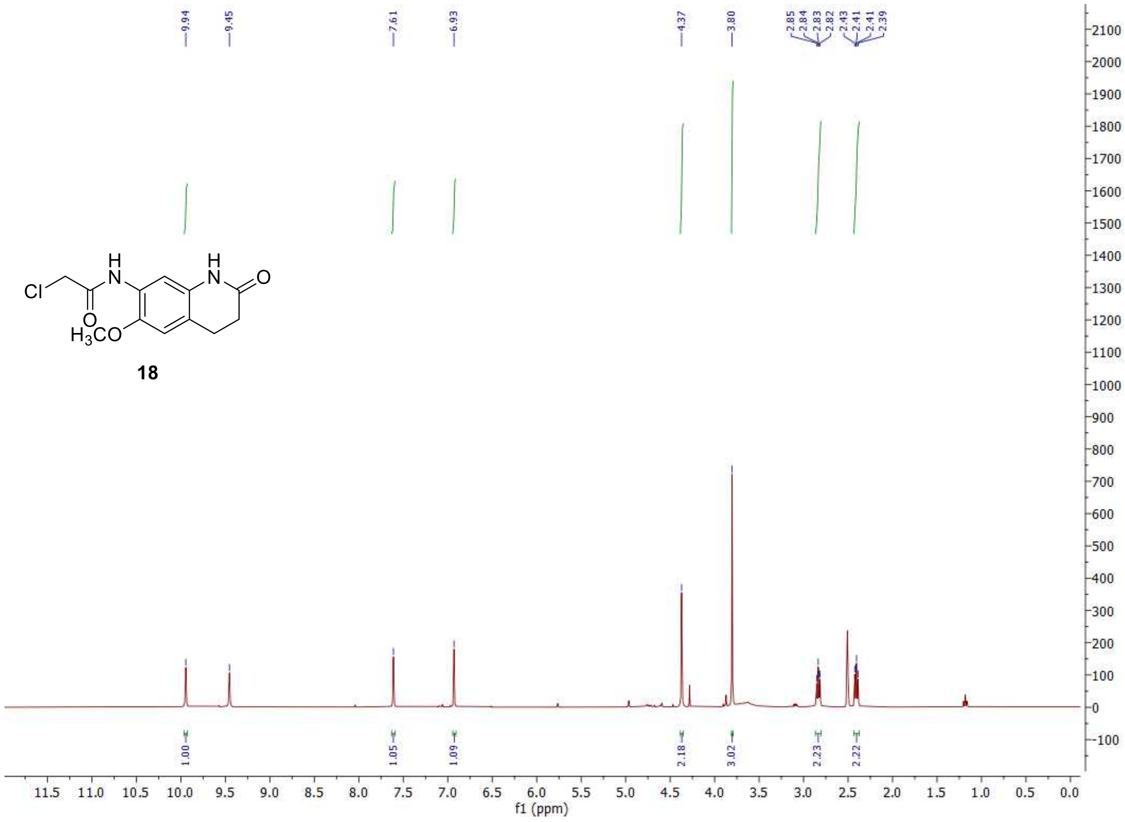
Cl
H
N
O
$H_3CO$
H
N
O
9.94
9.45
7.61
6.93
4.37
3.80
2.85
2.84
2.83
2.82
2.43
2.41
2.41
2.39
1.00
1.05
1.09
2.18
3.02
2.23
2.22
11.5 11.0 10.5 10.0 9.5 9.0 8.5 8.0 7.5 7.0 6.5 6.0 5.5 5.0 4.5 4.0 3.5 3.0 2.5 2.0 1.5 1.0 0.5 0.0
f1 (ppm)

**18**

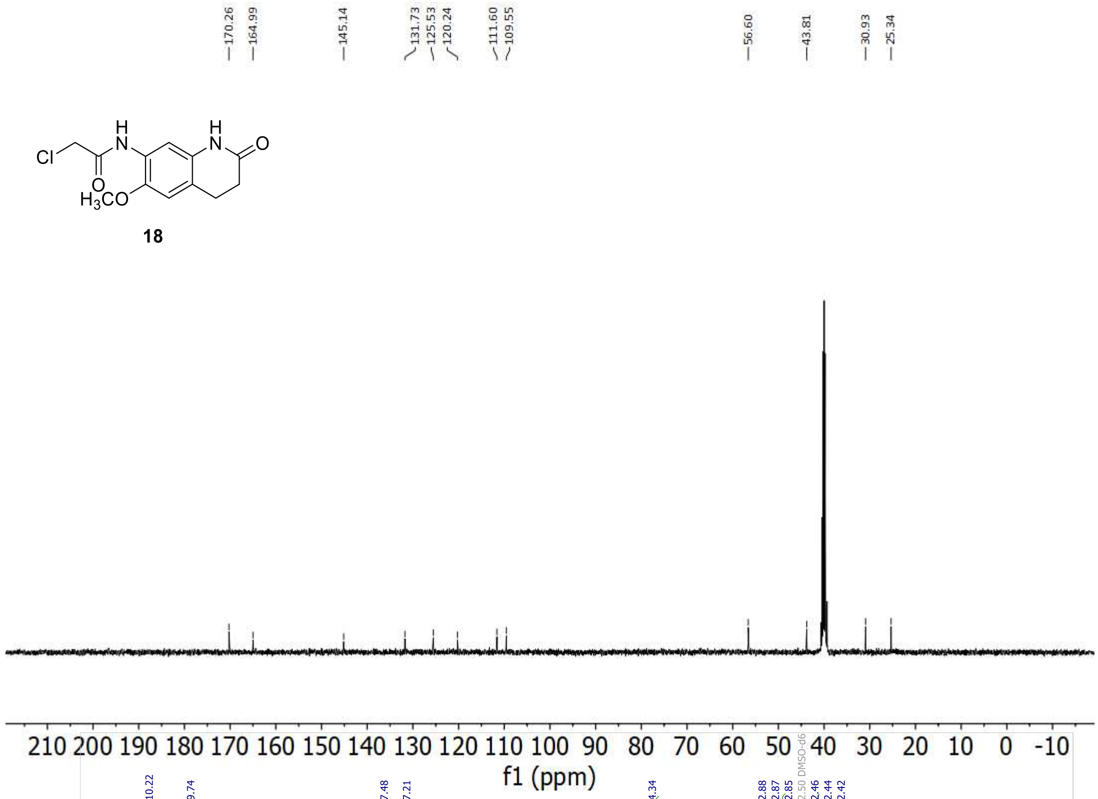


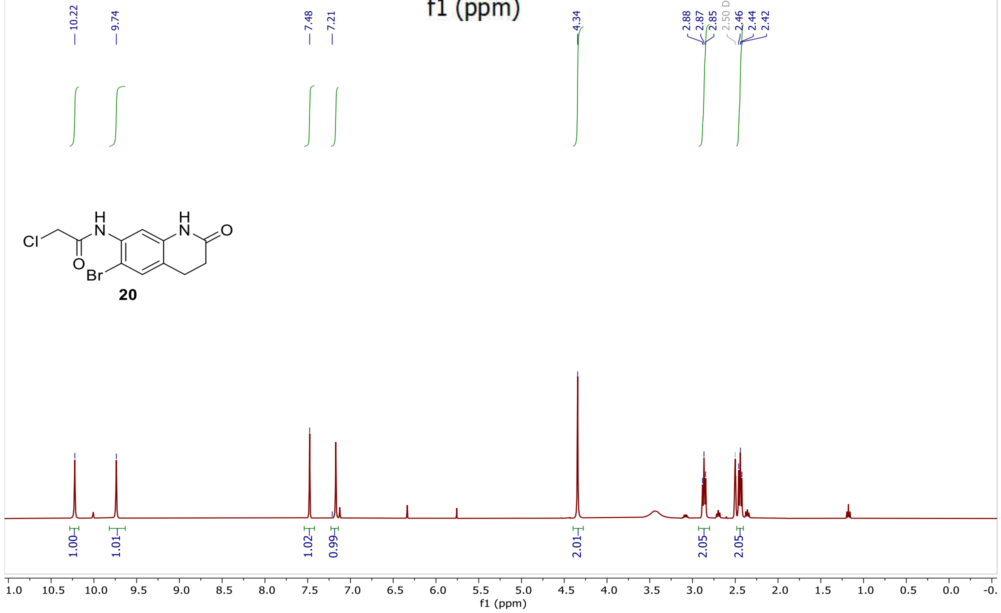


## Fluorescence based Mpro(3CL) activity assays

The ability of compounds **1**, **19** and **20** to inhibit Mpro was determined using SensoLyte® 520 SARS-CoV-2 3CL Protease Activity Assay Kit (Anaspec), following the manufacturer's instructions. Briefly, 40 µL diluted Mpro enzyme solution was added to a flat-bottom 96-well plate. 10 µL of varying concentrations of test compounds or GC 376 solution (an Mpro inhibitor) was

subsequently added to the enzyme solution. The mixed solutions were incubated at room temperature for 20 min. 50 μL of Mpro protease substrate solution was added to each well, followed by 30 min incubation at 37 °C. The Mpro activity was detected by measuring the fluorescence emission intensity $\lambda_{excitation}$=490 nm; $\lambda_{emission}$=520nm.

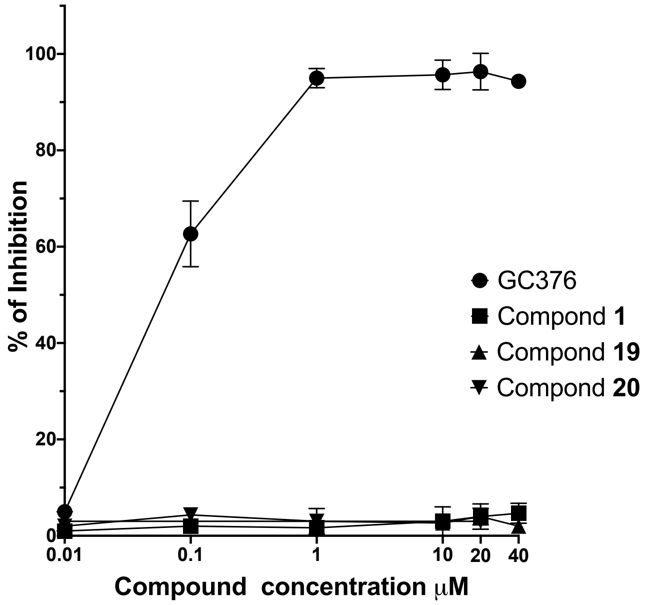


**Figure S5:** Compounds **1**, **19,** and **20** have no interaction with SARS-CoV-2 Mpro(3CL).

**Fluorescence based PLpro activity assay in the presence of dithioerythritol (DTT) and glutathione (GSH)**

PLpro inhibition assays were performed in the presence of 5 mM DTT and GSH to determine if compound **1** was active in the presence of thiols. PLpro protease activity was measured with peptide substrate x-x-G-G-AMC (Bachem) in the presence or absence of 5 mM DTT and GSH. PLpro was added into a black, flat-bottom 96-well plate at a final concentration of 50 nM with reaction buffer [150 mM NaCl, 20 mM HEPES, 0.1mg/mL BSA, pH 7.5] with or without 5 mM DTT or 5 mM GSH]. Compound **1** was added to the enzyme solution at concentrations ranging from 0-80 μM for 20 min at room temperature. The enzyme activity assay was initiated by mixing the x-x-G-G-AMC probe at a final 50 μM and incubating for another 20 min at room temperature. The reaction was ended by adding 1 μL of aqueous citric acid. The PLpro activity was detected by measuring the fluorescence emission intensity $\lambda_{excitation}$=360 nm; $\lambda_{emission}$=460nm.

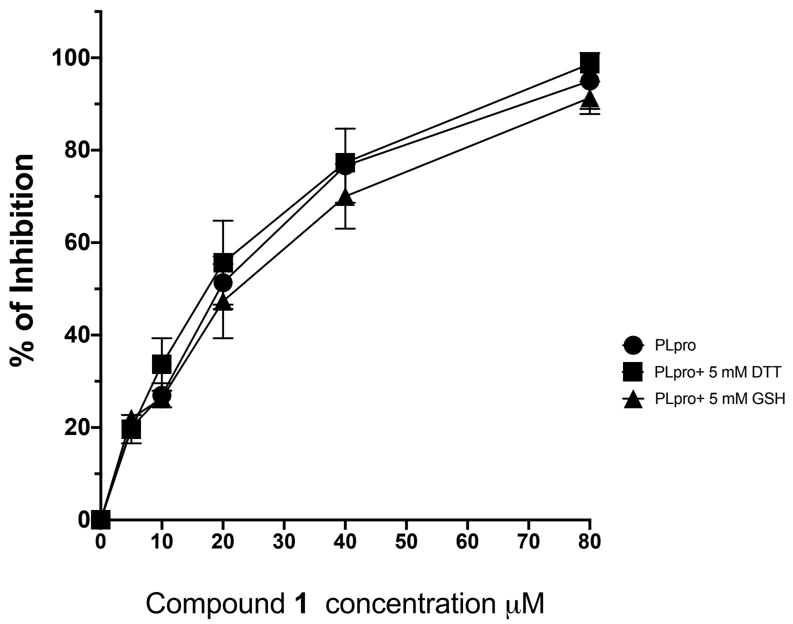


**Figure S6:** PLpro inhibition experiments with compound **1** in the presence of 5 mM DTT or 5 mM GSH.
